# Exploring Cinnamoyl-Substituted Mannopyranosides: Synthesis, Evaluation of Antimicrobial Properties, and Molecular Docking Studies Targeting H5N1 Influenza A Virus

**DOI:** 10.3390/molecules28248001

**Published:** 2023-12-07

**Authors:** Sabina Akter, Bader Y. Alhatlani, Emad M. Abdallah, Supriyo Saha, Jannatul Ferdous, Md Emdad Hossain, Ferdausi Ali, Sarkar M. A. Kawsar

**Affiliations:** 1 Laboratory of Carbohydrate and Nucleoside Chemistry (LCNC), Department of Chemistry, Faculty of Science, University of Chittagong, Chittagong 4331, Bangladesh; sabinaprimu@gmail.com (S.A.); jannat_080911@yahoo.com (J.F.); 2 Unit of Scientific Research, Applied College, Qassim University, Buraydah 52571, Saudi Arabia; 3 Department of Science Laboratories, College of Science and Arts, Qassim University, ArRass 51921, Saudi Arabia; 140208@qu.edu.sa; 4 Uttaranchal Institute of Pharmaceutical Sciences, Uttaranchal University, Dehradun 248007, Uttarakhand, India; supriyo9@gmail.com; 5 Wazed Miah Science Research Centre, Jahangirnagar University, Savar, Dhaka 1342, Bangladesh; emdad121542@gmail.com; 6 Department of Microbiology, Faculty of Biological Science, University of Chittagong, Chittagong 4331, Bangladesh; seema@cu.ac.bd

**Keywords:** antibacterial activity, antifungal, antiviral agents, mannopyranosides, molecular dynamics, FMO, MEP, ORSIS

## Abstract

The pursuit of innovative combinations for the development of novel antimicrobial and antiviral medications has garnered worldwide interest among scientists in recent times. Monosaccharides and their glycosides, such as methyl α-d-mannopyranoside derivatives, play a significant role in the potential treatment of viral respiratory pathologies. This study was undertaken to investigate and assess the synthesis and spectral characterization of methyl α-d-mannopyranoside derivatives **2**–**6**, incorporating various aliphatic and aromatic groups. The investigation encompassed comprehensive in vitro antimicrobial screening, examination of physicochemical properties, molecular docking analysis, molecular dynamics simulations, and pharmacokinetic predictions. A unimolar one-step cinnamoylation reaction was employed under controlled conditions to produce methyl 6-*O*-cinnamoyl-α-d-mannopyranoside **2**, demonstrating selectivity at the C-6 position. This represented a pivotal step in the development of potential antimicrobial derivatives based on methyl α-d-mannopyranoside. Subsequently, four additional methyl 6-*O*-cinnamoyl-α-d-mannopyranoside derivatives were synthesized with reasonably high yields. The chemical structures of these novel analogs were confirmed through a thorough analysis of their physicochemical properties, elemental composition, and spectroscopic data. In vitro antimicrobial assays were conducted against six bacterial strains and two fungal strains, revealing promising antifungal properties of these methyl α-d-mannopyranoside derivatives in comparison to their antibacterial activity. Moreover, cytotoxicity testing revealed that the compounds are less toxic. Further supporting these findings, molecular docking studies were performed against the H5N1 influenza A virus, indicating significant binding affinities and nonbonding interactions with the target protein 6VMZ. Notably, compounds **4** (−7.2) and **6** (−7.0) exhibited the highest binding affinities. Additionally, a 100 ns molecular dynamics simulation was conducted to assess the stability of the complex formed between the receptor 6VMZ and methyl α-d-mannopyranoside derivatives under in silico physiological conditions. The results revealed a stable conformation and binding pattern within the stimulating environment. In silico pharmacokinetic and toxicity assessments of the synthesized molecules were performed using Osiris software (version 2.9.1). Compounds **4** and **6** demonstrated favorable computational and pharmacological activities, albeit with a low drug score, possibly attributed to their higher molecular weight and irritancy. In conclusion, this study showcases the synthesis and evaluation of methyl α-d-mannopyranoside derivatives as promising candidates for antimicrobial and antifungal agents. Molecular docking and dynamics simulations, along with pharmacological predictions, contribute to our understanding of their potential therapeutic utility, although further research may be warranted to address certain pharmacological aspects.

## 1. Introduction

In 2023, there was growing global apprehension regarding the widespread emergence of antibiotic-resistant bacteria. This alarming trend is exacerbated by the notable absence of the development of new antibiotic classes, leading to what is commonly described as an “antibacterial crisis” [1]. Similarly, fungal infections are increasingly becoming a predominant source of morbidity and mortality among individuals with compromised immune systems. The rising incidence of mycoses can be attributed to the continual emergence of resistance to the antifungal drugs employed in clinical practice, compounded by the restricted diversity of available antifungal drug classes, which in turn constrains the spectrum of treatment options [2]. Furthermore, the situation is exacerbated by the fact that viral diseases stand as the predominant infectious diseases, accounting for the primary cause of global mortality. Notable among these viral diseases affecting humans are influenza, the common cold, hepatitis, chickenpox, Ebola, AIDS, hepatitis A virus, and COVID-19. These diseases have afflicted more than 80% of the world’s population, with a concerning absence of access to effective therapeutic interventions [3]. Influenza viruses have the capability to infect a wide range of vertebrate species, including humans, with influenza A, B, and C viruses being responsible for human infections. Their propensity for rapid mutation facilitates evasion of the host’s immune defenses. A historic testament to the devastating potential of influenza is the ‘Great Influenza’ pandemic of 1918, which remains unparalleled as the most severe recorded outbreak of infectious disease in human history. There is ongoing concern within the scientific community about the possibility of highly pathogenic avian influenza viruses, particularly those of the H5 and H7 subtypes, undergoing genetic changes that could lead to the emergence of pandemics of similar magnitude [4]. The highly virulent species H5N1 virus has mutated and spread over Asia, the Middle East, and Europe since its discovery. After the 2005 Qinghai Lake bird outbreak in western China, this pattern is noticeable [5]. Instead of seasonal influenza, H5N1 extends outside the respiratory tract. In addition, the virus inhibits cytotoxic T-cell function in vitro, lowering the host’s infection control. H5N1 influenza may potentially be caused by aberrant proinflammatory cytokine production [6]. Currently, three recommended antiviral drugs for treating the flu are oseltamivir (Tamiflu), zanamivir (Relenza), and peramivir (Rapivab). These drugs inhibit neuraminidase on the virus’s surface, preventing the release of viral particles from infected cells. They are effective against both influenzas A and B, but their impact is greatest when administered within 48 h of symptom onset. Timely diagnosis and treatment are crucial. These antivirals play a vital role in controlling influenza by treating infections and preventing severe complications such as bacterial pneumonia [7,8]. However, there is a pressing demand for novel strategies to mitigate influenza virus infections caused by H5N1 mutants, particularly within vulnerable patient populations and in the context of an influenza pandemic [9]. Recently, the Advisory Committee on Immunization Practices (ACIP) of the Centers for Disease Control and Prevention (CDC) and the American Academy of Family Physicians (AAFP) advocated for the annual administration of influenza vaccination to individuals aged six months and older, except those with contraindications [10]. However, there are some concerns regarding the potential reduction in vaccine effectiveness against influenza viruses, compounded by a greater decline in efficacy following repeated vaccinations [11]. In the foreseeable future, prognosticators anticipate the potential emergence of a global influenza pandemic [12].

Methyl α-d-mannopyranoside is a simple monosaccharide sugar occurring naturally as mannose polymers in every plant tissue. Dietary exposure to alpha methyl mannoside results from the breakdown of mannose polymers found in many plant-based foods, with the highest concentrations found in guar gum, a common food additive used to improve the consistency of cooked foods, dairy products, meats, spices, and coffee. Alpha methyl mannoside does not cause mutations and has a minimal toxicity profile [13]. Furthermore, the use of methyl α-d-mannopyranoside has been observed in the process of mannosylation of lipid nanoparticles (LNPs) designed for the purpose of vaccine or medicine delivery, specifically targeting antigen-presenting cells (APCs) by means of mannose receptors [14,15]. Moreover, the investigation of selective acylation and the evaluation of antimicrobial activity in monosaccharide compounds have revealed that the introduction of heterocyclic aromatic rings with electron-attracting or electron-donating groups significantly enhances the biological characteristics compared to the initial molecules [16,17,18,19,20]. The growing importance of carbohydrate derivatives as highly promising antibacterial and therapeutic agents has garnered interest in the synthesis of derivatives of mannopyranoside. In the present study, regioselective cinnamoylation of methyl α-d-mannopyranoside (**1**) with antimicrobial and in silico studies, which may be scientific investigations directed toward the development of pharmaceutical interventions for influenza, is of paramount significance.

## 2. Results

### 2.1. Chemistry

**Figure 1:**
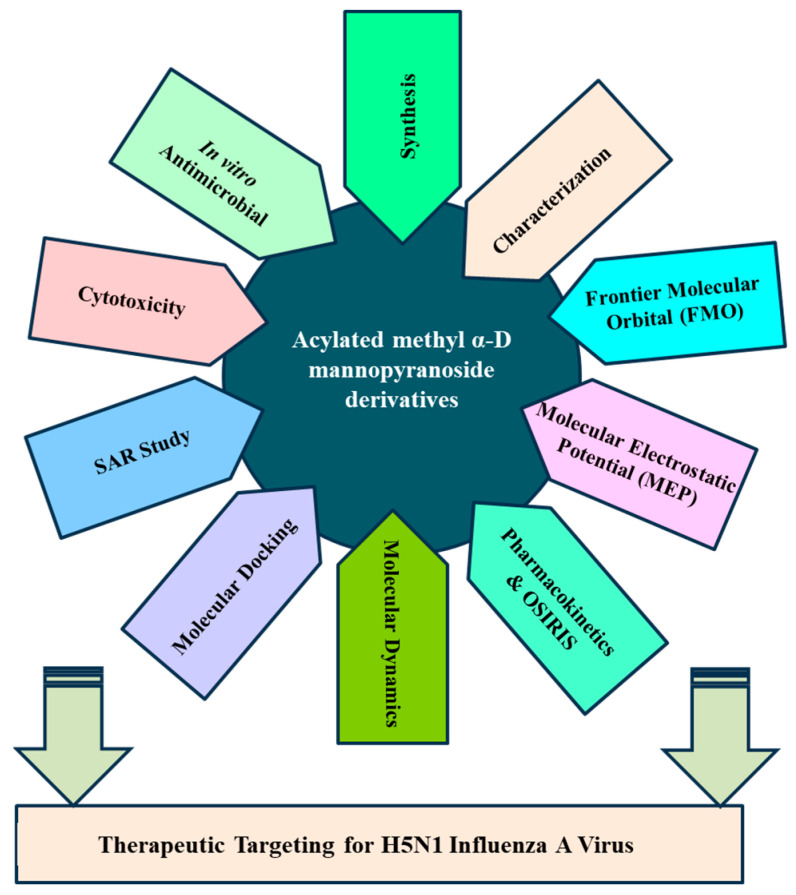
Illustrates the workflow of the present study.

**Scheme 1:**
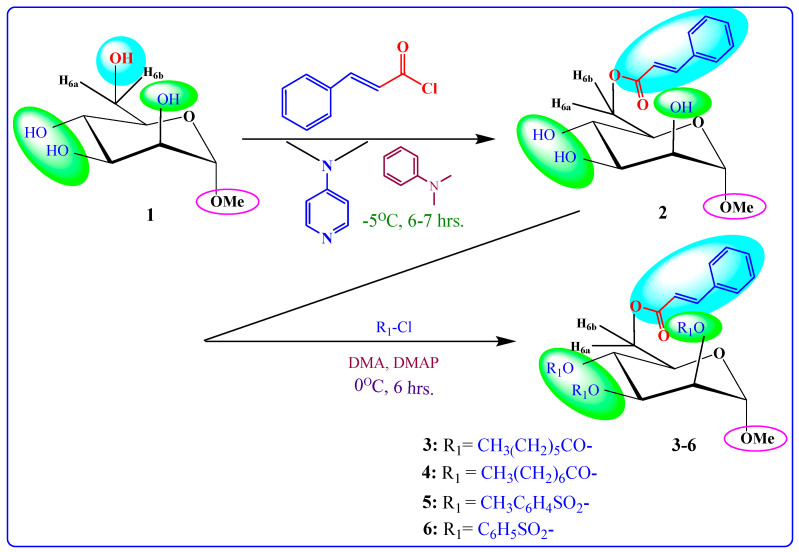
Reagents and conditions: cinnamoyl chloride; DMA (*N*,*N*-dimethylaniline) (as solvent); DMAP (4-dimethylaminopyridine) (as catalyst); −5 °C; 6–7 h; R_1_-Cl = several acyl halides; 0 °C to rt; stirrer for 6 h (**3**–**6**).

This study aimed to conduct regioselective cinnamoylation of methyl α-d-mannopyranoside (**1**) using direct acylation. The work plan is schematically shown in Figure 1. From the products, several acylating agents were used to synthesis derivatives (Scheme 1). Analysis of FTIR and ^1^H-NMR spectra revealed the acylation product’s structure (Table 1).

### 2.2. Characterization

Our initial effort (Scheme 1) was to treat methyl α-d-mannopyranoside (**1**) with cinnamoyl chloride as an acylating agent in anhydrous *N*,*N*-dimethylaniline (DMA) as a solvent and DMAP (4-dimethylaminopyridine) as a catalyst at −5 °C, and after the usual work-up, compound (**2**) was obtained in excellent yields (80.75%). This compound was sufficiently pure for use in the next stages. Its FTIR spectrum showed absorption bands at 1681 cm^−1^ (for -CO stretching), 3380–3425 cm^−1^ (br.) (for -OH stretching) and 1621 cm^−1^ (for -CH=CH- stretching). The ^1^H-NMR spectrum analysis distinctly confirmed the formation of a mono-substitution product. This confirmation was evident from the presence of a single one-proton doublet peak at δ 7.51 (1H, d, *J* = 16.1 Hz, corresponding to PhCH=CHCO-) and another one-proton doublet peak at δ 6.49 (1H, d, *J* = 16.0 Hz, representing PhCH=CHCO-), signifying the presence of a lone cinnamoyl group within the molecular structure [21]. Furthermore, the spectrum exhibited a two-proton multiplet at δ 7.72 (appearing as a multiplet, denoted as Ar-H) and a three-proton multiplet at δ 7.38 (also appearing as a multiplet, denoted as Ar-H), attributed to the protons in the aromatic ring of the cinnamoyl group in the molecule. The shift of the C-6 protons of the mannopyranose rest to higher chemical shifts, specifically δ 4.85 (appearing as a multiplet, denoted as H-6a) and δ 4.81 (appearing as a multiplet, denoted as H-6b), compared to their initial values (~4.00 ppm in compound **6**), indicated the attachment of the cinnamoyl group at the less hindered and more reactive position, namely, position 6. The remainder of the FTIR and ^1^H-NMR spectra consistently matched the structure assigned to methyl 6-*O*-cinnamoyl-α-d-mannopyranoside (**2**) [22].

To further validate the structure of the cinnamoyl derivative (**2**), we proceeded with its transformation into the heptanoyl (**3**) and octanoyl (**4**) derivatives. This involved the reaction of compound **2** with an excess of heptanoyl chloride in *N*,*N*-dimethylaniline (DMA) as a solvent, followed by the standard work-up procedure and purification using silica gel column chromatography. The FTIR spectrum of compound **3** displayed notable absorption peaks at 1701 cm^−1^, corresponding to carbonyl (-CO) stretching, and at 1622 cm^−1^, indicative of (-CH=CH-) stretching. Notably, there was an absence of hydroxyl stretching in this spectrum. The incorporation of three octanoyl groups into the molecule was evidenced by the emergence of specific signals in the ^1^H-NMR spectrum. These signals included two six-proton multiplets at δ 2.38 (3 × CH_3_(CH_2_)_5_C**H**_2_CO-), δ 1.61 (3 × CH_3_(CH_2_**)**_4_C**H**_2_CH_2_CO-), a twenty-four-proton multiplet at δ 1.26 (3 × CH_3_(C**H_2_**)_4_(CH_2_)_2_CO-), and a nine-proton multiplet at δ 0.90 (3 × C**H**_3_(CH_2_)_6_CO-). These findings strongly suggested the presence of three heptanoyl groups attached to the triol molecule **2**. Additionally, the resonances of C-2, C-3, and C-4 were observed at δ 4.72 (as d, J = 3.1 Hz), δ 4.61 (as dd, J = 3.0 and 9.0 Hz), and δ 4.58 (as t, J = 9.0 Hz), exhibiting a downfield shift compared to the triol (**2**) values. This shift indicated the incorporation of the three heptanoyl groups at positions 2, 3, and 4. Upon comprehensive analysis of the ^1^H-NMR and FTIR spectra, the structure of heptanoate was unequivocally confirmed as methyl 6-*O*-cinnamoyl-2,3,4-tri-*O*-heptanoyl-α-d-mannopyranoside (**3**). Subsequently, the identical cinnamoyl derivative **2** underwent a transformation into the octanoyl derivative (**4**). All other FTIR and ^1^H-NMR signals were consistent with their expected positions, further substantiating the structure of this compound as methyl 6-*O*-cinnamoyl-2,3,4-tri-*O*-octanoyl-α-d-mannopyranoside (**4**).

Upon subjecting the cinnamoyl derivative (**2**) to a reaction with an equimolecular amount of *p*-toluenesulfonyl chloride at freezing temperatures, followed by conventional work-up steps and column chromatography, it yielded the tri-*O*-*p*-toluenesulfonyl derivative (**5**). The ^1^H-NMR spectrum displayed characteristic peaks at δ 8.02 (3 ×2H, multiplet, denoted as Ar-H) and δ 7.96 (3 ×2H, multiplet, denoted as Ar-H), confirming the presence of three *p*-toluenesulfonyl groups within the compound. All other signals in both the FTIR and ^1^H-NMR spectra were consistent with the proposed structure, confirming the identity of this compound as methyl 6-*O*-cinnamoyl-2,3,4-tri-*O*-*p*-toluenesulfonyl-α-d-mannopyranoside (**5**). Motivated by these promising outcomes, we employed benzenesulfonyl chloride as the subsequent acylating agent. The treatment of compound **2** with benzenesulfonyl chloride in DMF, followed by the same work-up procedures and column chromatography, yielded compound **6** in a needle form, attaining an 81.27% yield. The ^1^H-NMR spectrum exhibited peaks at δ 7.56 (6H, multiplet), δ 7.45 (3H, multiplet), and δ 7.27 (6H, multiplet), corresponding to the protons of three phenyl groups. Upon a comprehensive analysis of the FTIR and ^1^H-NMR spectra, along with reference to similar derivatives described previously, the structure of this compound was confidently determined as methyl 2,3,4-tri-*O*-benzenesulfonyl-6-*O*-cinnamoyl-α-d-mannopyranoside (**6**).

### 2.3. Antibacterial Potentiality

**Figure 2:**
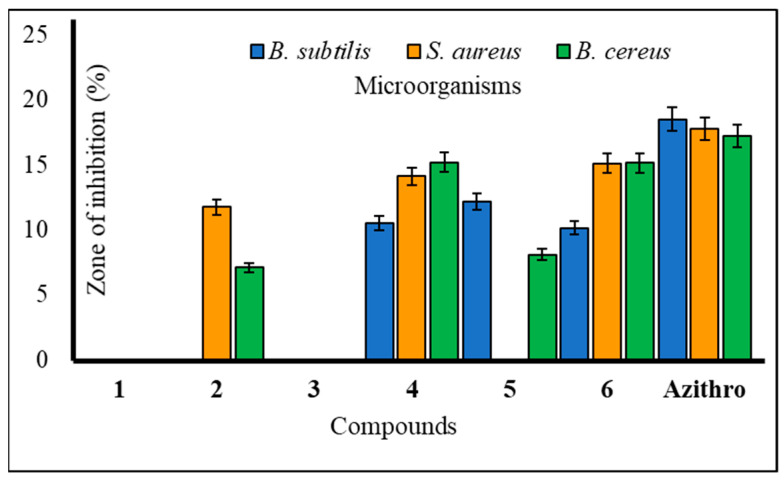
Zone of inhibition observed against Gram-positive bacteria by compounds **1**–**6**.

**Figure 3:**
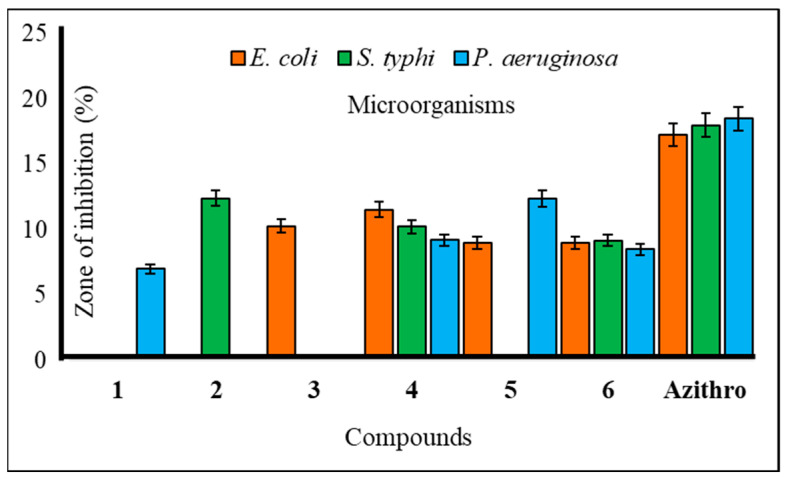
Zone of inhibition observed against Gram-negative bacteria by compounds **1**–**6**.

The utilization of carbohydrate analogs represents an innovative approach due to their potential to impact genomic processes, thereby disrupting critical transcription or replication mechanisms necessary for microbial survival. Since pathogens typically lack alternative pathways for these fundamental metabolic processes, nucleoside analogs effectively function as antimicrobial agents by inhibiting these essential pathways. A series of synthesized mannopyranoside derivatives (**2**–**6**) were subjected to in vitro antimicrobial assessments against various strains of both Gram-positive bacteria (*Bacillus subtilis*, *Staphylococcus aureus*, and *Bacillus cereus*) and Gram-negative bacteria (*Escherichia coli*, *Salmonella typhi*, and *Pseudomonas aeruginosa*). The evaluation methods encompassed the disk diffusion technique and the broth microdilution method for determining the minimum inhibitory concentration (MIC) and minimum bactericidal concentration (MBC), following established protocols [23]. Notably, derivative **5** displayed a variable zone of inhibition, with a measured zone of 12.15 ± 0.7 mm against *Bacillus subtilis* and 12.36 ± 0.2 mm against *Pseudomonas aeruginosa*. Compound **3**, on the other hand, demonstrated a moderate zone of inhibition against a single Gram-negative bacterium, *Escherichia coli* (10.25 ± 0.2 mm). Based on these observations, the hierarchy of Gram-positive antibacterial activity among the mannopyranoside analogs can be ranked as follows: **6** > **4** > **5** > **2** > **3**. The preliminary antibacterial results are presented in Table 2. Notably, compound **4** demonstrated the highest inhibition zone against *Bacillus cereus* (15.17 ± 0.4 mm) and *Staphylococcus aureus* (14.10 ± 0.7 mm) (Figure 2). Compound **6** also exhibited a significant inhibition zone against *S. aureus* (15.12 ± 0.1 mm) and *B. cereus* (15.15 ± 0.3 mm). Furthermore, derivative **2** displayed moderate inhibition zones against Gram-positive *B. cereus* (11.75 ± 0.3 mm) and Gram-negative *S. typhi* (12.41 ± 0.3 mm) (Figure 3). The assessment of antibacterial efficacy was categorized in accordance with established criteria from prior research. Specifically, a diameter measuring 10 mm or less was considered indicative of weak activity, while a range between greater than 10 mm and 15 mm was suggestive of moderate activity. An antibacterial effect exceeding a diameter of 15 mm was indicative of high activity [24,25]. In the scientific literature, benzoyl derivatives bearing cinnamoyl substitutions exhibit significant antibacterial properties against some Gram-positive and Gram-negative bacterial strains [26]. A range of amino-3-acetoxymethyl cephalosporins with cinnamoyl substitutions exhibited selective antibacterial activity specifically targeting Gram-positive bacteria. These compounds hold promise as potential candidates for the development of anti-MRSA (methicillin-resistant *Staphylococcus aureus*) agents, with potential for further enhancements through structural modifications [27]. Methicillin-resistant *Staphylococcus aureus* (MRSA) is a type of bacteria that is resistant to several antibiotics.

**Figure 4:**
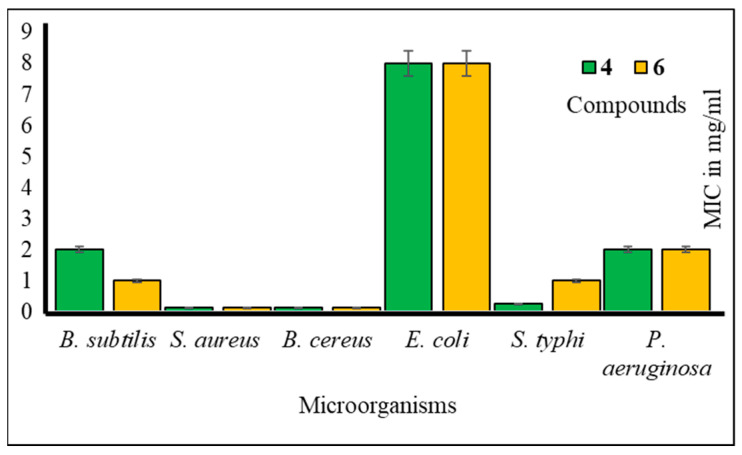
MIC values of compounds **4** and **6** against tested pathogens.

**Figure 5:**
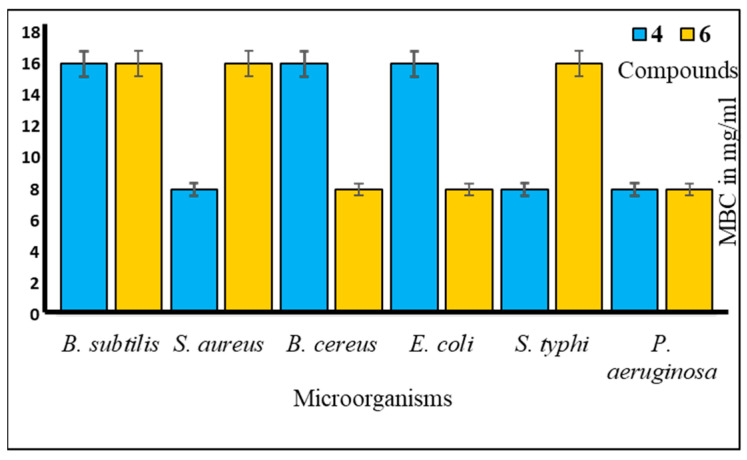
MBC values of compounds **4** and **6** against tested pathogens.

Furthermore, to assess the antibacterial efficacy against pathogenic bacteria, we determined both the minimal inhibitory concentration (MIC) and minimal bactericidal concentration (MBC) values for the most potent mannopyranoside derivatives. These results are presented in Figure 4 and Figure 5. Among the tested derivatives, mannopyranoside compounds **4** and **6** exhibited the most robust antibacterial effects, with MIC values ranging from 0.125 to 8.0 mg/mL. Compound **4** displayed activity against all tested bacterial strains, showcasing its marked effectiveness, particularly against both *Staphylococcus aureus* and *Bacillus cereus*, with an MIC of 0.125 mg/mL. Interestingly, compound **6** demonstrated impressive activity against a majority of the tested pathogens, achieving notably low MIC values, with its most potent activity observed against *Salmonella typhi* (0.125 mg/mL). Regarding the MBC values, both derivatives **4** and **6** exhibited the lowest MBC value of 8.00 mg/mL against *Bacillus subtilis* and *Salmonella typhi*. Conversely, the highest MBC value of 16.00 mg/mL was recorded for these derivatives against *Escherichia coli*, *Bacillus subtilis*, and *Pseudomonas aeruginosa*. The MBC values for these compounds against the other tested organisms fell within this range of 8.00–16.00 mg/mL, indicating their bactericidal activity. The MIC test determines the in vitro susceptibility or resistance of specific bacterial strains to drugs. It plays a critical role in guiding antibiotic treatment, identifying resistance, optimizing dosages, and supporting drug development against pathogens [28]. However, the MBC test is of paramount importance, as it discerns the minimum drug concentration necessary for bacterial eradication, thereby informing treatment strategies, evaluating drug resistance, and refining therapeutic protocols. Additionally, it serves as a valuable tool for concurrent assessment of the antibacterial efficacy of multiple agents [29].

### 2.4. Antifungal Susceptibility

**Figure 6:**
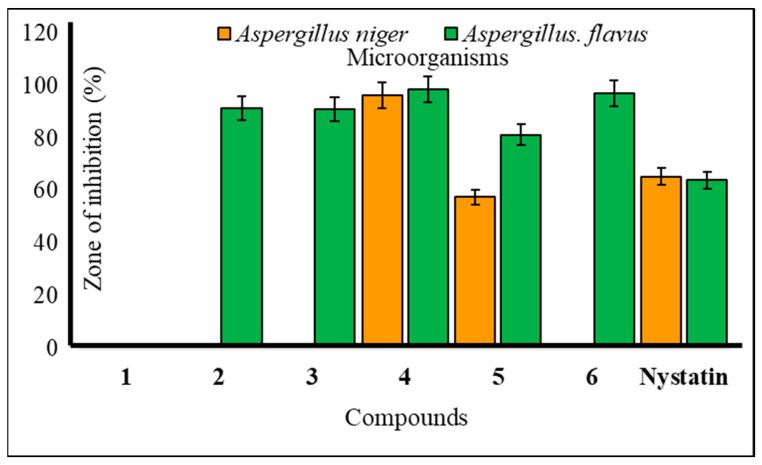
Mean percentage of mycelial inhibition of the tested compounds against *A. niger* and *A. flavus*.

The majority of methyl α-d mannopyranoside (**1**) derivatives exhibited notable inhibition of mycelial growth in both *A. niger* and *A. flavus* (Table 3). Compound **4** displayed significant inhibition at 96.22 ± 1.1% against *A. niger* and 98.47 ± 1.0% against *A. flavus* in antifungal assessments, surpassing the standard antibiotic, nystatin. Compounds 2–6 also demonstrated remarkable mycelial growth inhibition against *A. flavus* at levels of 91.34 ± 1.0%, 91.12 ± 1.0%, 98.47 ± 1.0%, 81.30 ± 1.0%, and 97.02 ± 1.1%, respectively, in the mycelial growth test. Compound **4** exhibited promising mycelial growth inhibition against both *A. niger* and *A. flavus* (Figure 6). Additionally, compounds **2**, **3**, and **6** displayed strong inhibition against *A. flavus* but did not affect *A. niger*. This suggests that mannopyranoside acylation enhances antimicrobial activity. The results indicate that the presence of various acyl groups, including cinnamoyl, heptanoyl, octanoyl, *p*-toluenesulfonyl, and benzenesulfonyl groups, significantly improves the antimicrobial activity of methyl α-d-mannopyranoside (**1**) derivatives. Our results align with previous publications; hexanoylation of methyl α-d-mannopyranoside yielded 6-*O*-hexanoate, which was transformed into five 2,3,4-tri-*O*-acyl esters. These esters exhibited superior antifungal activity, as determined by PASS and in vitro assessment. Quantum chemical analysis, molecular docking, and ADMET predictions supported their potential as antifungal agents. Structure–activity relationship (SAR) analysis revealed that hexanoylation combined with acetic, caprylic, or lauric chains showed the best antifungal properties [30]. Methyl α-d-mannopyranoside, including acylated forms, was tested for antifungal activity against phytopathogenic fungi. Many compounds surpassed the standard antibiotic nystatin in effectiveness [31].

### 2.5. Cytotoxic Activity of Mannopyranoside Compounds

**Figure 7:**
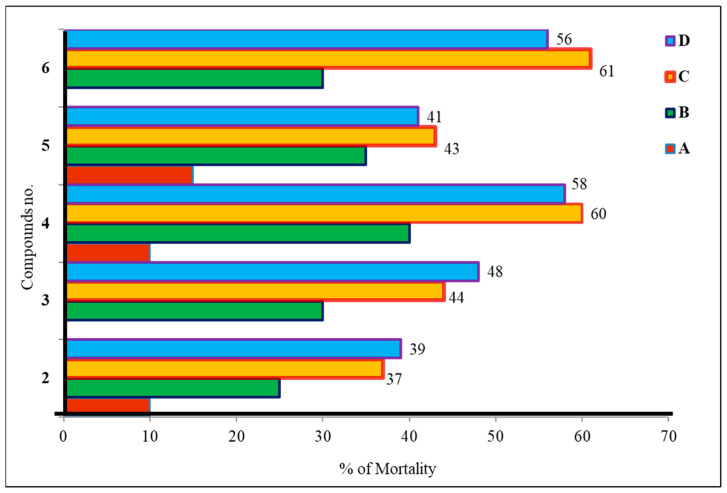
Cytotoxic activity of mannopyranoside derivatives (**2**–**6**). The tested mannopyranoside derivatives were prepared at four different concentrations in the vials; vials containing 4, 8, 16, and 32 µL were labeled as **A**, **B**, **C**, and **D**, respectively.

According to the brine shrimp lethality test (Figure 7), the produced mannopyranoside derivatives (**2**–**6**) are cytotoxic [32]. Both 24 and 48 h shrimp mortality rates are shown. As the study shows, longer alkyl chains and phenyl rings increase hydrophobicity and cytotoxicity [33]. According to data analysis, compound **2** (methyl 6-*O*-cinnamoyl-α-d-mannopyranoside) had the lowest toxicity and had a death rate of 27.07%. Compounds **4** and **6** showed higher toxicity, resulting in death ranges of 36.10–37.03%. Benzoyl derivatives are less cytotoxic than alkanoyl derivatives per this observation. Furthermore, alkyl chain derivative concentration increases cytotoxicity.

### 2.6. Structure–Activity Relationship Study

**Figure 8:**
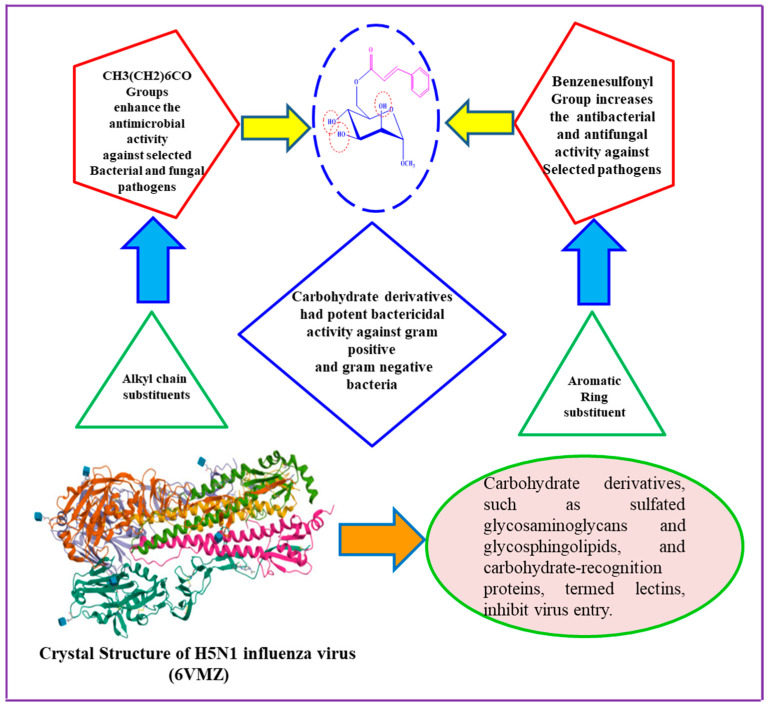
Structure–activity relationship study (SAR) of the synthesized derivatives.

**Figure 9:**
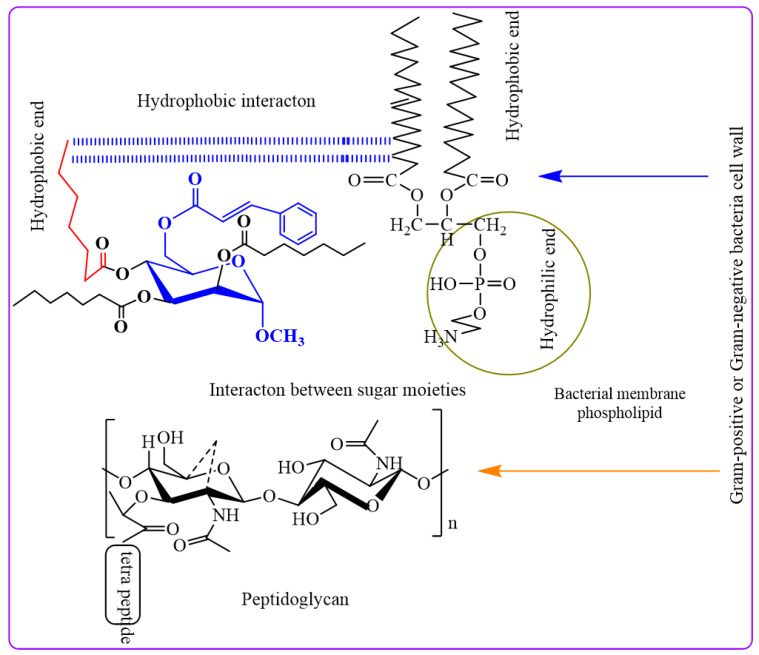
SAR study of the mannopyranoside compounds with bacterial pathogens.

The development of antimicrobial agents is of utmost importance due to the escalating multidrug resistance in common pathogens and the emergence of new infections. These agents, typically comprising diverse five- and six-membered heterocyclic molecules, are vital in the metabolic processes of all living cells. Moreover, condensed ring systems have garnered significant attention for their diverse physiological activities and success as privileged medicinal scaffolds. Given that monosaccharides play pivotal roles in fundamental cellular metabolic functions, it is unsurprising that mannopyranoside derivatives exhibit the capability to target various enzymes involved in bacterial peptidoglycan biosynthesis, fungal chitin biosynthesis, and protein synthesis. Structure–activity relationship (SAR) analysis, as depicted in Figure 8 and Figure 9, is crucial in elucidating the mechanisms underlying the antibacterial activity of mannopyranoside derivatives. The SAR analysis, based on the antimicrobial activity results presented in Table 2 and Table 3, reveals that modifications to the mannopyranoside skeleton significantly impact antibacterial activity. Interestingly, native mannopyranoside exhibited no activity against pathogenic bacteria, highlighting the profound influence of structural changes on antibacterial efficacy. Notably, benzene-substituted acyl groups, particularly attached benzenesulphonyl groups, enhance the activity of the parent compound. In contrast, for straight-chain acyl groups, there is a discernible trend of increasing activity with elongation of the acyl chain length. This order of influence holds true for both Gram-positive and Gram-negative bacteria. The structure–activity relationship (SAR) is a critical concept in chemistry and pharmacology. It is the study of the relationship between the chemical structure of a compound and its biological or pharmacological activity. SAR plays a crucial role in drug discovery and development, environmental risk assessment, and the understanding of toxicology [34].

The peptidoglycan layer of Gram-negative bacteria, which is enclosed in an LPS-containing membrane, explains these phenomena. LPS causes selective permeability and hinders diffusion. This outer layer blocks certain things from entering. Cyclic acyl chains in carbohydrates, which are inherent to lipid-like bacterial membranes, can communicate without water. When hydrophobic interactions impair membrane permeability, bacteria perish [35].

In our investigation, it was observed that higher activity was exhibited by fused benzenesulfonyl moieties compared to *p*-toluenesulfonyl, while derivatives containing heptanoyl were found to be less potent than those containing octanoyl. Notably, higher concentrations of the synthesized compounds were found to be needed to inhibit the growth of Gram-negative bacteria (MIC values in the range of 2 to 8 mg/L) compared to their Gram-positive counterparts. This differential behavior can be attributed to the distinct structural characteristics of the cell walls in Gram-positive and Gram-negative bacteria. An outer membrane surrounding the peptidoglycan layer, coated with lipopolysaccharides (LPS), is possessed by Gram-negative bacteria. This outer membrane is acted upon as a formidable barrier, limiting the diffusion of substances. The periplasmic region in Gram-negative bacteria harbors hydrolytic enzymes responsible for neutralizing environmental chemicals [36]. Gram-positive bacteria exhibit a thick, hydrophilic, porous structure without an outer membrane, rendering them more permeable. Generally, Gram-positive bacteria are more susceptible to synthetic derivatives [37]. Furthermore, the incorporation of heptanoyl and octanoyl groups progressively increased the hydrophobicity of the methyl α-d-mannopyranoside derivatives. Hydrophobicity is a critical factor for bioactivity, as it can impact membrane integrity and permeability [38]. Studies have suggested that the efficacy of aliphatic alcohols correlates with their lipid solubility, driven by hydrophobic interactions with lipid membrane regions [39]. Similarly, hydrophobic interactions may occur between the acyl chains of methyl α-d-mannopyranoside derivatives and the lipid-like components of bacterial membranes. These interactions disrupt membrane permeability, ultimately leading to bacterial death.

### 2.7. Molecular Docking Simulation

**Figure 10:**
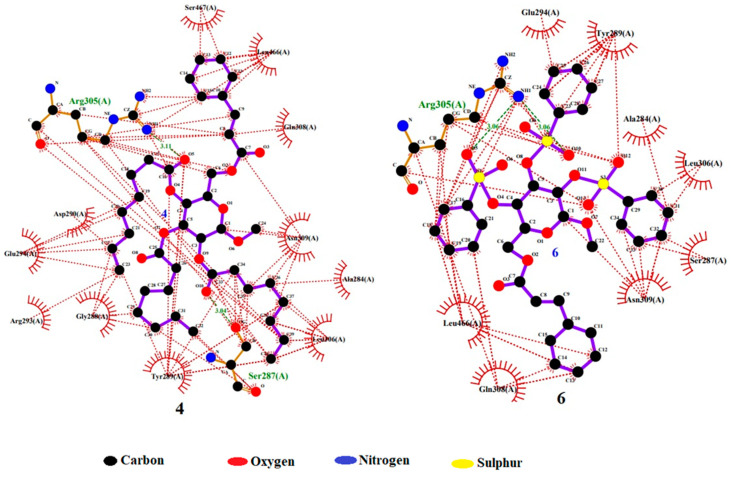
Docking interaction pose of compounds **4** and **6** with 6VMZ.

According to the results, compound **4** displayed the highest dock score of (−) 7.2 kcal/mol, while compound **6** exhibited a dock score of (−) 7.0 kcal/mol. In the docking interaction of compound **4**, notable interactions included the hydrogen bonding of the hydroxyl group of SER 287 and the guanidine NH of ARG 305 with the carbonyl oxygen of the -OC=O(CH_2_)_6_CH_3_ groups attached at the C3 and C4 positions of the structure, with bond distances of 3.04 Å and 3.11 Å, respectively. Additionally, hydrophobic interactions occurred between ALA 284, GLY 288, TYR 289, ASP 290, ARG 293, GLU 294, LEU 306, GLN 308, ASN 309, LEU 466, and SER 467 with the -C_6_H_5_ and -(CH_2_)_6_CH_3_ groups of compounds [40]. In the case of compound **6**, a hydrogen bond interaction was observed between the guanidine NH of ARG 305 and the oxygen of the -OSO_2_C_6_H_5_ groups attached at the C4 and C5 positions of the structure, with a bond distance of 3.06 Å. Additionally, hydrophobic interactions were established between ALA 284, SER 287, TYR 289, GLU 294, LEU 306, GLN 308, ASN 309, and LEU 466 and the -C6H5, S1, S2, O5, O7, O10, and O12 groups of compound **6** (Table 4). These findings confirm the successful docking of these compounds with the receptor, as illustrated in Figure 10 [41].

### 2.8. Molecular Dynamics

**Figure 11:**
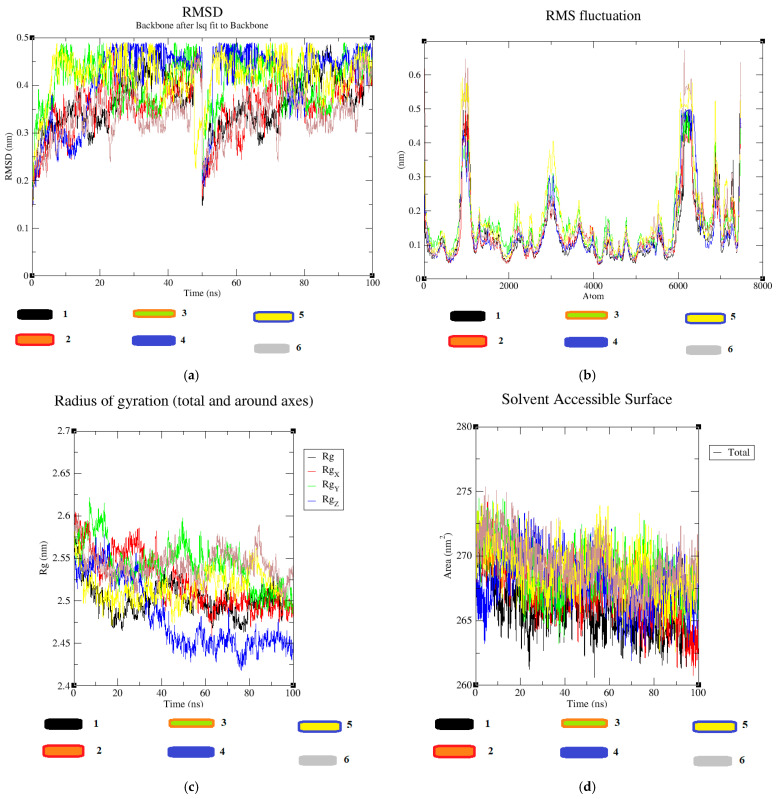
(**a**–**d**) MD simulation data of the synthesized compound 4-6 VMZ complexes.

Based on the molecular dynamic simulation data for molecules (**1**–**6**) in comparison to 6VMZ, the average root mean square deviation (RMSD) values were as follows: 0.36, 0.35, 0.40, 0.41, 0.41, and 0.33. Figure 11a illustrates that the RMSD values reach a relatively stable plateau. Regarding the root mean square fluctuation (RMSF) data, the values fluctuated within the ranges of 0.043–0.49, 0.041–0.49, 0.056–0.49, 0.045–0.49, 0.05–0.59, and 0.046–0.67 for the ligand–receptor complexes. Figure 11b displays three noticeable fluctuations occurring in approximately 1000 atoms, 3000 atoms, and 6000 atoms based on the RMSF data, although these fluctuations were not highly significant. Analysis of the radius of gyration data revealed that the gyration values decreased from their initial values for all the ligand–protein complexes, ranging from 2.46 to 2.57, 2.45 to 2.60, 2.48 to 2.62, 2.41 to 2.57, 2.47 to 2.58, and 2.49 to 2.60 for compounds (**1**–**6**) in complexes with the protein, respectively. A lower radius of gyration indicates greater structural stability. Figure 11c highlights that among all the complex structures, the 4–6VMZ docked complex exhibited the highest stability. The average values for the solvent-accessible surface area (SASA) of the complexes were 265.87 nm^2^, 267.25 nm^2^, 268.78 nm^2^, 267.97 nm^2^, 268.95 nm^2^, and 269.22 nm^2^, respectively. Figure 11d illustrates that SASA values remained relatively constant throughout the analysis, confirming their positive impact on binding energy [42].

### 2.9. MM/PBSA Analysis

A good interaction in terms of molecular mechanics between drug–receptor interactions such as bonded energy, electrostatic energy, van der Waals interaction, polar energy, and SASA energy determined by MM/PBSA (molecular mechanics/Poisson–Boltzmann surface area) analysis. The results from MM/PBSA analysis revealed the following maximum binding energies: compound **4** exhibited the highest with −198.29 kJ/mol, followed by compound **6** with −27.87 kJ/mol, and compound **1** with −24.49 kJ/mol (Table 5). In the compound **4**–**6** VMZ receptor complex, specific energy components were observed as follows: van der Waals energy (−0.18 kJ/mol), electrostatic energy (−158.34 kJ/mol), polar solvation energy (−39.93 kJ/mol), and solvent-accessible surface area (SASA) energy (+0.033 kJ/mol). Compound **6** exhibited the maximum van der Waals energy (−104.331 kJ/mol), while compound 4 displayed the highest electrostatic energy (−158.34 kJ/mol), polar solvation energy (−39.93 kJ/mol), and SASA energy (−13.26 kJ/mol) within this context. Based on computational data, compounds **4** and **6** demonstrated promising outcomes among the developed compounds.

### 2.10. Frontier Molecular Orbital (FMO) Analysis

Compounds **1**–**6** were selected for frontier molecular orbital analysis. The HOMO orbital energy (eV) values of **1**–**6** were −7.34, −6.61, −6.77, −6.83, −6.61, and −6.12, respectively. The LUMO orbital energy (eV) values of **1**–**6** were −0.217, −2.04, −2.50, −2.93, −2.61, and −2.85, respectively. A smaller distance between the highest occupied and lowest unoccupied molecular orbital confirmed the chemical reactivity. Compound **6** showed a minimum HOMO-LUMO energy distance of 3.27 eV, followed by compounds 4, 5, 3, and 2 with HOMO-LUMO energy distances of 3.90 eV, 4.00 eV, 4.27 eV, and 4.57 eV, respectively [43]. (Table 6 and Figure S1 (Supplementary Materials)).

### 2.11. Molecular Electrostatic Potential (MEP) Investigation

In the case of compound **1**, the maximum portions of the structure comprised green- to yellow-colored mild nucleophilic–electrophilic regions. In the case of compound **2**, the maximum portion of the structure was green, and only the third and fourth carbons of the terminal phenyl and the sixth carbon of the terminal phenyl showed possible areas for electrophilic and nucleophilic attacks, respectively. In the case of compound **3**, the red-colored electrophilic region and blue-colored nucleophilic region were focused on the ester group and alkyl chain of the heptanoate group, respectively. In the case of compound **4**, the red-colored electrophilic region and blue-colored nucleophilic region were focused on the ester group and alkyl chain of the octanoate side chain, respectively. For compound **5**, the red-colored electrophilic region and blue-colored nucleophilic region were focused on the ester group of the phenyl-2-en ester and the sulfonyl group of the 4-methyl phenyl sulfonyloxy group, respectively. In the case of compound **6**, the red-colored electrophilic region and blue-colored nucleophilic region were focused on the ester group of the phenyl-2-en ester and the sulfonyl group of the phenyl sulfonyloxy group, respectively (Figure S2).

### 2.12. Pharmacokinetics Properties

Considering the ADMET (absorption, distribution, metabolism, excretion, and toxicity) profiling data, compound **2** demonstrates the highest water solubility among the listed compounds, boasting a log value of −1.673. In contrast, compounds **3**, **4**, **5**, and **6** exhibit progressively lower water solubility values (Table 7). However, compounds **3** and **4** display the highest Caco-2 permeability, registering values of 0.784 and 0.748, respectively. Compound **2**, on the other hand, exhibits lower Caco-2 permeability at 0.31. Compounds **5** and **6** show negative values, indicating potential challenges in traversing intestinal cells. However, compounds **5** and **6** demonstrated relatively high values for intestinal absorption at 85.269% and 84.047%, respectively. Compound **4** also exhibits a favorable absorption rate of 77.499%. In terms of skin permeability, compounds **3–6** showcase similar values, ranging from −2.729 to −2.735. None of the compounds exhibited CYP2C19 inhibitory activity. Total clearance values span from 0.175 to 1.609, with compounds **3** and **4** displaying the highest clearance rates. All compounds exhibit negative values for blood–brain barrier (BBB) and central nervous system (CNS) permeability, suggesting potential challenges in crossing these barriers. In summary, compounds **3** and **4** demonstrate favorable characteristics for intestinal absorption and distribution within the body but also exhibit higher clearance rates. None of the compounds function as CYP2C19 inhibitors, which can impact drug metabolism (Table 8). Furthermore, all compounds face challenges in breaching the blood–brain barrier and accessing the central nervous system. The selection of a compound for further drug development hinges on the specific objectives and requisites of the drug development process, considering factors such as absorption, metabolism, and distribution.

### 2.13. OSIRIS Data

**Figure 12:**
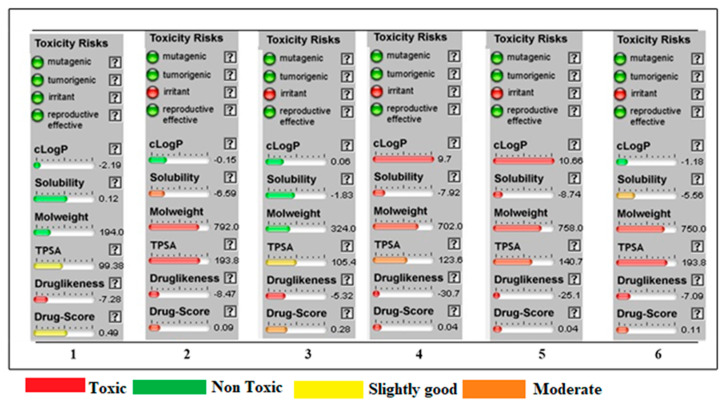
OSIRIS data of the synthesized compounds.

The data indicate that among the compounds evaluated, only compound **1** exhibited no indications of mutagenicity, tumorigenicity, irritancy, or reproductive toxicity. While compounds **4** and **6** demonstrated favorable computational and pharmacological properties, it is noteworthy that their OSIRIS data yielded drug scores of 0.04 and 0.11, potentially attributed to their higher molecular weights, which could contribute to irritancy concerns (Table 9 and Figure 12). Synthesized molecules showed the presence of non-mutagenicity, non-tumourigenicity, and non-reproductive toxicity behaviors. Only compound **1** was non-irritant in nature.

## 3. Discussion

This study comprehensively examined five previously synthesized methyl α-d-mannopyranoside derivatives (**2**–**6**) through synthesis, characterization, in vitro antimicrobial assays, and in silico computational approaches, including molecular docking and molecular dynamics. In antimicrobial testing, compound **5** exhibited variable inhibition zones, with measurements of 12.15 ± 0.7 mm against *Bacillus subtilis* and 12.36 ± 0.2 mm against *Pseudomonas aeruginosa*. Compound **3** displayed moderate inhibition (10.25 ± 0.2 mm) against *Escherichia coli*. Gram-positive antibacterial activity ranked as **6** ˃ **4** ˃ **5** ˃ **2** ˃ **3**. Compounds **4** and **6** demonstrated remarkable antimicrobial effects, with MIC values ranging from 0.125 to 8.0 mg/L. Compound **4** showed broad-spectrum activity, with the most significant potency against *Staphylococcus aureus* and *Bacillus cereus* (MIC 0.125 mg/L). Compound **6** exhibited excellent activity against *Salmonella typhi* (MIC 0.125 mg/L). MBC values varied from 8.00 mg/L for compounds **4** and **6** (against *B. subtilis* and *S. typhi*) to 16.00 mg/L (against *E. coli*, *B. subtilis*, and *P. aeruginosa*). Molecular docking interactions between synthesized molecules and 6VMZ receptor confirmed that compounds **4** and **6** showed maximum docking scores of −7.2 kcal/mol and −7.0 kcal/mol, respectively. Compound **4** showed hydrogen bond interactions of 3.04 Å and 3.11 Å distances between the hydroxyl group of SER 287 and the guanidine NH group of ARG 305 with the carbonyl oxygen of -OC=O(CH_2_)_6_CH_3_ group attached with C3 and C4 positions of the structure, respectively. Also, some hydrophobic interactions were also formed between ALA 284, GLY 288, TYR 289, ASP 290, ARG 293, GLU 294, LEU 306, GLN 308, ASN 309, LEU 466, and SER 467 amino acids with phenyl and heptyl groups of compound **4** [40]. In compound **6**, a hydrogen bond of 3.06 Å distance formed between the guanidine NH of ARG 305 and the oxygen of the -OSO_2_C_6_H_5_ groups present at the C4 and C5 positions of the molecule. Additionally, hydrophobic interactions were established between ALA 284, SER 287, TYR 289, GLU 294, LEU 306, GLN 308, ASN 309, and LEU 466 and the -C6H5, S1, S2, O5, O7, O10, and O12 groups of compound **6**. The MD simulation data showed that all molecules attained almost static RMSD values, and the RMSD values ranged between 0.2 and 0.5. The RMS fluctuations showed some fluctuations near 1000 and 7000 atoms up to 0.6 nm. The radius of the gyration value is directly correlated with the structural stability. A high value means less stability and vice versa. Lower-range values of the radius of gyration represent higher stability. The radius of gyration values near 2.5 nm. SASA values were almost constant throughout the analysis, which confirmed that SASA values positively impacted the binding energy. Here, lower SASA values in the complexes showed higher stability. The SASA values of all the molecules attained a lower value near 250 nm^2^, which confirmed that they were highly stable complexes. Molecular dynamics simulations revealed stable conformational changes, minimal fluctuations in RMSD and RMSF values, and improved structural stability, as indicated by the radius of gyration data. Surface area calculations (SASA) validated their positive impact on binding energy. Electrophilicity, electronegativity, hardness, chemical softness, and electronic transition between electron-rich and electron-deficient species of a chemical structure are explained by FMO. Distance between HOMO and LUMO energies is inversely proportional with drug–receptor interaction, which means high distance, less stability, and vice versa [44]. Electronegativity values reflect the reactivity of a chemical structure. Among all the synthesized compounds, compounds **4** and **6** showed maximum electronegative and electrophilic natures, respectively. These two molecules are designated as the most reactive molecules. Benzene sulfonyl and 3-phenylprop-2-enoic acid groups of compounds **6** and **4** are expressed as HOMO and LUMO orbital, respectively [45]. The reactive and stability of the synthesized molecules were maintained this way from compound **6,** followed by compounds **4**, **5**, **3**, and **2**. Molecular electrostatic potential data confirmed the importance of different functional groups associated with electrophilic and nucleophilic attack regions. In the in silico computational approach, binding scores, stability of protein–ligand complexes, antiviral predictions, physicochemical properties, bioactivity, and pharmacokinetic properties against the H5N1 influenza A virus were assessed. Mannopyranoside compounds (**2**–**6**) showed improved binding affinity compared to the parent methyl α-d-mannopyranoside. Derivatives with longer carbon chains or aromatic rings exhibited enhanced binding affinities. Aliphatic compounds (**3**–**4**) displayed increased binding scores, while aromatic compounds (**5** and **6**) also exhibited notable binding affinities. This suggests that large aliphatic chains may enhance binding and biological efficacy. Compounds **4** and **6** emerged as potential agents against H5N1 influenza and antibacterial candidates.

## 4. Materials and Methods

### 4.1. Material and Equipment

All extracted organic products, obtained using either chloroform (CHCl_3_) or dichloromethane (CH_2_Cl_2_), were subjected to drying with anhydrous sodium sulfate (Na_2_SO_4_) or magnesium sulfate (MgSO_4_) before concentration. Solvent evaporation occurred under reduced pressure utilizing a VV–1 type vacuum rotary evaporator (BUCHI, Flawil, Switzerland) equipped with a BUCHI-461 (Flawil, Switzerland) water bath, maintaining a temperature below 40 °C. Melting points were determined using an electrothermal melting point apparatus (Fisher Scientific, Hampton, NH, USA), and the reported values are uncorrected for solid and thoroughly dried compounds. Infrared (IR) spectra were recorded either as CHCl_3_ solutions or KBr discs, and for films, measurements were conducted at the Chemistry Department, University of Chittagong, Bangladesh, utilizing an IR Affinity Fourier-transform infrared spectrophotometer (Shimadzu, Kyoto, Japan). Nuclear magnetic resonance (NMR) spectra (400/100 MHz) were recorded in CDCl_3_, employing tetramethyl silane (TMS) as an internal standard, utilizing a Bruker DPX-400 spectrometer (400 MHz) at the Bangladesh Council of Scientific and Industrial Research (BCSIR) Laboratories, Dhaka, Bangladesh. For synthesized compounds, chromatogram development was achieved by using an appropriate spraying agent, particularly 1% H_2_SO_4_, followed by gentle heating to 200 °C on a hot plate to visualize distinct spots on thin-layer chromatography (TLC) plates.

### 4.2. Synthesis

*Methyl 6-O-cinnamoyl-α-d-mannopyranoside (**2**)*

Methyl α-d-mannopyranoside (1) (100 mg, 0.515 mmol) was dissolved in 3 mL of anhydrous *N*,*N*-dimethylaniline (DMA), DMAP (4-dimethylaminopyridine), and cooled to −5 °C. Cinnamoyl chloride (0.0925 mg, 1.1 molar equivalent) was added to the solution, and the reaction mixture was stirred continuously for 6 h at 0 °C. Subsequently, the reaction mixture was left to stand at room temperature with continuous stirring overnight. The progress of the reaction was monitored using thin-layer chromatography (TLC) with a solvent system of methanol–chloroform (2:5), which indicated complete conversion of the starting material into a single product (*R_f_* = 0.52). The mixture was subjected to silica gel column chromatography with a methanol–chloroform elution ratio of 1:4 to facilitate the purification of the target compound (**2**) as a solid. Recrystallization was performed with EtOAc-*n*-hexane, and needles of cinnamoyl derivative (2) (134.8 mg) were obtained. Yield 80.75%, m.p.: 88–89 °C. FTIR: ν_max_ 3380–3425 (br., -OH), 1681 (C=O), 1628 (-CH=CH-) cm**^−^**^1^. ^1^H-NMR (400 MHz, CDCl_3_): δ_H_ (ppm) 7.72 (2H, m, Ar-H), 7.51 (1H, d, *J* = 16.1 Hz, PhC*H*=CHCO-), 7.38 (3H, m, Ar-H), 6.49 (1H, d, *J* = 16.0 Hz, PhCH=C*H*CO-), 4.85 (1H, m, H-6a), 4.81 (1H, m, H-6b), 4.14 (1H, s, H-1), 4.0 (1H, d, *J* = 3.1 Hz, H-2), 3.89 (1H, t, *J* = 9.1 Hz, H-4), 3.72 (1H, dd, *J* = 3.1 and 9.3 Hz, H-3), 3.44 (1H, m, H-5), 3.34 (3H, s, 1-OC*H*_3_). ^13^C NMR (100 MHz, CDCl_3_): δ_C_ 165.84 (C_6_H_5_CH=CH*C*O-), 150.58 (C_6_H_5_*C*H=CHCO-), 132.99, 132.01, 129.20 (×2), 129.06 (×2) (*C*_6_H_5_CH=CHCO-), 122.26 (C_6_H_5_CH=*C*HCO-), 97.09 (C-1), 72.92 (C-2), 71.32 (C-4), 70.62 (C-3), 69.38 (C-5), 63.05 (C-6), 55.16 (1-O*C*H_3_). LC–MS [M + 1]^+^ 325.27. Calcd. for C_16_H_20_O_7_:C = 59.21, H = 6.22; found: C = 59.22, H = 6.20%.

#### General Procedure for the Synthesis of Cinnamoyl Derivatives **3**–**6**

*Methyl 2,3,4-tri-O-heptanoyl-6-O-cinnamoyl-α-d-mannopyranoside (**3**)*

A stirred solution of compound (2) (113.9 mg, 0.351 mmol) in dry *N*,*N*-dimethylaniline (DMA) (3 mL) and DMAP (4-dimethylaminopyridine) catalyst was maintained at 0 °C and treated with heptanoyl chloride (0.27 mL, 5 molar equivalents). The low temperature was sustained using ice and common salt for 6 h with continuous stirring. The progress of the reaction was monitored by thin-layer chromatography (TLC) using a methanol–chloroform (2:5) solvent system, which indicated complete conversion of the starting material into a single product (*R_f_* = 0.52). The work-up and purification were performed as described earlier, including silica gel column chromatography with methanol–chloroform (2:5) as the eluant. This process yielded title compound (**3**) (143 mg) in the form of a crystalline solid, followed by recrystallization (EtOAc-*n*-hexane). Yield 61.64%, m.p.: 101–102 °C, recrystallization EtOAc-*n*-hexane. FTIR: ν_max_ 1701 (C=O), 1622 (-CH=CH-) cm**^−^**^1^. ^1^H-NMR (400 MHz, CDCl_3_): δ_H_ (ppm) 7.78 (2H, m, Ar-H), 7.55 (1H, d, *J* = 16.0 Hz, PhC*H*=CHCO-), 7.39 (3H, m, Ar-H), 6.52 (1H, d, *J* = 16.2 Hz, PhCH=C*H*CO-), 4.86 (1H, s, H-1), 4.72 (1H, d, *J* = 3.1 Hz, H-2), 4.61 (1H, dd, *J* = 3.0 and 9.0 Hz, H-3), 4.58 (1H, t, *J* = 9.0 Hz, H-4), 4.20 (1H, m, H-6a), 4.16 (1H, m, H-6b), 3.94 (1H, m, H-5), 3.41 (3H, s, 1-OC*H*_3_), 2.40 {6H, m, 3 × CH_3_(CH_2_)_4_C*H*_2_CO-}, 1.65 {6H, m, 3 × CH_3_(CH_2_)_3_C*H*_2_CH_2_CO-}, 1.32 {18H, m, 3 × CH_3_(C*H*_2_)_3_CH_2_CH_2_CO-}, 0.90 {9H, m, 3 × C*H*_3_(CH_2_)_5_CO-}. ^13^C NMR (100 MHz, CDCl_3_): δ_C_ 175.50, 174.73, 173.79 {3 × CH_3_(CH_2_)_5_*C*O-}, 165.76 (C_6_H_5_CH=CH*C*O-), 150.32 (C_6_H_5_*C*H=CHCO-), 132.89, 132.11, 129.26 (×2), 129.34 (×2) (*C*_6_H_5_CH=CHCO-), 122.55 (C_6_H_5_CH=*C*HCO-), 97.11 (C-1), 72.76 (C-2), 71.11 (C-4), 70.34 (C-3), 69.31 (C-5), 63.11 (C-6), 55.04 (1-O*C*H_3_), 34.09, 34.05, 34.02, 33.82, 31.21, 31.12, 31.10, 24.77, 24.53, 24.44, 24.41, 22.27, 22.22, 22.20, 21.87 {3 × CH_3_(*C*H_2_)_5_CO-}, 13.83, 13.81, 13.80 {3 × *C*H_3_(CH_2_)_5_CO-}. LC–MS [M + 1]^+^ 661.636. Calcd. for C_37_H_56_O_10_: C = 67.23, H = 8.54; found: C = 67.25, H = 8.56%. Similar reaction and purification methods were employed to synthesize an octanoyl derivative (**4**) (218 mg as needles), *p*-toluenesulfonyl derivative (**5**) (348.9 mg as needles), and benzenesulfonyl derivative (**6**) (232.4 mg as needles).

*Methyl 6-O-cinnamoyl-2,3,4-tri-O-octanoyl-α-d-mannopyranoside (**4**)*

Yield 74.10%, m.p.: 97–98 °C, recrystallization EtOAc-*n*-hexane. FTIR: ν_max_ 1700 (C=O), 1625 (-CH=CH-) cm**^−^**^1^. ^1^H-NMR (400 MHz, CDCl_3_): δ_H_ (ppm)7.70 (2H, m, Ar-H), 7.54 (1H, d, *J* = 16.3 Hz, PhC*H*=CHCO-), 7.42 (3H, m, Ar-H), 6.50 (1H, d, *J* = 16.1 Hz, PhCH=C*H*CO-), 5.16 (1H, d, J = 3.4 Hz, H-1), 5.01 (1H, m, H-2), 4.86 (1H, t, J = 9.2 Hz, H-3), 4.80 (1H, m, H-4), 4.13 (1H, dd, *J* = 5.1 and 12.1 Hz, H-6a), 4.01 (1H, m, H-6b), 3.95 (1H, m, H-5), 3.41 (3H, s, 1-OC*H*_3_), 2.38 {6H, m, 3 × CH_3_(CH_2_)_5_C*H*_2_CO-}, 1.61 {6H, m, 3 × CH_3_(CH_2_)_4_C*H*_2_CH_2_CO-}, 1.26 {24H, m, 3 × CH_3_(C*H*_2_)_4_(CH_2_)_2_CO-}, 0.90 {9H, m, 3 × C*H*_3_(CH_2_)_6_CO-}. ^13^C NMR (100 MHz, CDCl_3_): δ_C_ 171.0, 170.89, 170.04 {3 × CH_3_(CH_2_)_6_*C*O-}, 165.77 (C_6_H_5_CH=CH*C*O-), 150.51 (C_6_H_5_*C*H=CHCO-), 132.32, 132.15, 129.27 (×2), 129.32 (×2) (*C*_6_H_5_CH=CHCO-), 122.15 (C_6_H_5_CH=*C*HCO-), 97.11 (C-1), 72.22 (C-2), 71.17 (C-4), 70.88 (C-3), 69.56 (C-5), 63.09 (C-6), 55.14 (1-O*C*H_3_), 34.21, 34.11 (×3), 32.0 (×3), 31.05 (×3), 25.13, 22.61 (×2), 21.45 (×2), 20.07 (×3) {3 × CH_3_(*C*H_2_)_6_CO-}, 14.15, 14.09, 14.01 {3 × *C*H_3_(CH_2_)_6_CO-}. LC–MS [M + 1]^+^ 703.726. Calcd. for C_40_H_62_O_10_: C = 68.31, H = 8.89; found: C = 68.29, H = 8.88%.

*Methyl 2,3,4-tri-O-p-toluenesulfonyl-6-O-cinnamoyl-α-d-mannopyranoside (**5**)*

Yield 86.71%, m.p.: 110–111 °C, recrystallization EtOAc-*n*-hexane. FTIR: ν_max_ 1707 (C=O), 1626 (-CH=CH-) cm**^−^**^1^. ^1^H-NMR (400 MHz, CDCl_3_): δ_H_ (ppm) 8.02 (3 × 2H, m, Ar-H), 7.96 (3 × 2H, m, Ar-H), 7.74 (2H, m, Ar-H), 7.50 (1H, d, *J* = 16.3 Hz, PhC*H*=CHCO-), 7.44 (3H, m, Ar-H), 6.58 (1H, d, *J* = 16.2 Hz, PhCH=C*H*CO-), 5.04 (1H, d, *J* = 3.5 Hz, H-1), 4.92 (1H, d, *J* = 3.6 Hz, H-2), 4.80 (1H, dd, *J* = 3.4 and 9.0 Hz, H-3), 4.64 (1H, t, *J* = 9.1 Hz, H-4), 4.50 (1H, m, H-6a), 4.10 (1H, m, H-6b), 4.01 (1H, m, H-5), 3.41 (3H, s, 1-OC*H*_3_). ^13^C NMR (100 MHz, CDCl_3_): δ_C_ 165.22 (C_6_H_5_CH=CH*C*O-), 144.20, 143.26 (×3), 138.49 (×3), 128.25 (×3), 128.24 (×3), 125.45 (×3), 20.69, 20.66 {3 × *p*-CH_3_*C*_6_H_4_SO_2_-}, 150.71 (C_6_H_5_*C*H=CHCO-), 132.63, 132.41, 129.56 (×2), 129.11 (×2) (*C*_6_H_5_CH=CHCO-), 122.32 (C_6_H_5_CH=*C*HCO-), 97.25 (C-1), 72.11 (C-2), 71.32 (C-4), 70.71 (C-3), 69.49 (C-5), 63.13 (C-6), 55.46 (1-O*C*H_3_), 14.20, 14.11, 14.04 {3 × *p*-CH_3_C_6_H_4_SO_2-_}. LC–MS [M + 1]^+^ 787.696. Calcd. for C_34_H_38_O_13_S_3_: C = 54.86, H = 5.34; found: C = 54.87, H = 5.36%.

*Methyl 6-O-cinnamoyl-2,3,4-tri-O-benzenesulfonyl-α-d-mannopyranoside (**6**)*

Yield: 81.27%, m.p.: 104–105 °C, recrystallization EtOAc-*n*-hexane. FTIR: ν_max_ 1702 (C=O), 1620 (-CH=CH-), 1363 (-SO_2_) cm**^−^**^1^. ^1^H-NMR (400 MHz, CDCl_3_): δ_H_ (ppm) 7.87 (2H, m, Ar-H), 7.56 (3 × 2H, m, 3 × Ar-H), 7.51 (1H, d, *J* = 16.1 Hz, PhC*H*=CHCO-), 7.45 (3 × 1H, m, 3 × Ar-H), 7.39 (3H, m, Ar-H), 7.27 (3 × 2H, m, 3×Ar-H), 6.49 (1H, d, *J* = 16.0 Hz, PhCH=C*H*CO-), 4.91 (1H, s, H-1), 4.82 (1H, d, *J*= 3.6 Hz, H-2), 4.80 (1H, dd, *J* = 3.3 and 9.0 Hz, H-3), 4.78 (1H, t, *J* = 9.2 Hz, H-4), 4.09 (1H, m, H-6a), 4.0 (1H, m, H-6b), 3.51 (1H, m, H-5), 3.31 (3H, s, 1-OC*H*_3_). ^13^C NMR (100 MHz, CDCl_3_): δ_C_ 165.84 (C_6_H_5_CH=CH*C*O-), 150.58 (C_6_H_5_*C*H=CHCO-), 144.35, 144.28, 144.07, 135.40, 135.17, 135.02, 129.79 (×3), 129.11 (×3), 126.90 (×3), 126.45 (×3) (×3*C*_6_H_5_SO_2_-), 132.99, 132.01, 129.20 (×2), 129.06 (×2) (*C*_6_H_5_CH=CHCO-), 122.26 (C_6_H_5_CH=*C*HCO-), 97.09 (C-1), 72.92 (C-2), 71.32 (C-4), 70.62 (C-3), 69.38 (C-5), 63.05 (C-6), 55.16 (1-O*C*H_3_),. LC–MS [M + 1]^+^ 745.606. Calcd. for C_34_H_32_O_13_S_13_: C = 54.79, H = 4.33; found: C = 54.81, H = 4.35%.

### 4.3. In Vitro Antimicrobial Activity Test

#### 4.3.1. Test Microorganisms

In this study, a total of eight microbial strains were employed, comprising three Gram-positive bacteria (*Staphylococcus aureus*, *Bacillus subtilis*, and *Bacillus cereus*), three Gram-negative bacteria (*Escherichia coli*, *Salmonella Typhi*, and *Pseudomonas aeruginosa*), and two fungi (*Aspergillus niger* and *Aspergillus flavus*). The microbial cultures were obtained from the Microbial Laboratory within the Department of Microbiology at the University of Chittagong, Bangladesh.

#### 4.3.2. Antibacterial Activity Evaluation

The synthesized compounds underwent antibacterial assessments using the standard disk diffusion assay in accordance with CLSI guidelines [46]. The preparation of the inoculum involved suspending test bacteria in sterile normal saline (0.9%), which was obtained from plates using a sterile inoculating loop. Adjustments to the suspension density were made by comparing it to the McFarland 0.5 standard. Subsequently, the test bacteria were swabbed onto plates containing Mueller–Hinton agar (MHA). Using an Eppendorf pipette, 10 µL of the test compounds were applied to filter paper discs (6 mm, Hi-Media) and positioned in the agar medium. The plates containing the seeded bacteria were incubated at 35 ± 2 °C for 24 h to facilitate the growth of test organisms. Results were quantified in terms of the diameter of the inhibition zones in millimeters. Each experiment was replicated three times, and the mean was calculated. Azithromycin discs (Incepta Pharma Ltd., Dhaka, Bangladesh) served as a standard antibiotic, acting as a positive control and undergoing evaluation alongside the tested compounds under identical conditions.

#### 4.3.3. Determination of MIC and MBC

The minimum inhibitory concentration (MIC) was evaluated using the microdilution method [47]. Serial two-fold dilutions of the synthesized compounds were prepared, covering concentrations ranging from 8.0 to 0.25 mg/mL in Muellesr–Hinton broth, supplemented with 5% dimethyl sulfoxide (DMSO). According to our preliminary experiments, bacterial growth remains unaffected by 5.0% DMSO. Subsequently, each well containing 100 μL of the serially diluted compound had 5 µL of a calibrated microbial suspension added, and 95 μL of sterile Luria–Bertani broth was introduced into all wells. A negative control, comprising all components without the bacterial suspension, was also included.

Following the designated overnight incubation at 35 °C, 20 mL of 2 mg/mL INT (*p*-iodonitrotetrazolium chloride) was introduced into all microplates. The plates underwent an additional 30 min incubation period, during which the presence of bacterial growth was indicated by the appearance of a purple-red color, resulting from the reduction of INT into formazan.

The determination of the minimum inhibitory concentration (MIC) involved identifying the lowest concentration of the compound capable of inhibiting bacterial growth after 24 h of exposure to the synthesized compound. The evaluation of the minimum bactericidal concentration (MBC) involved sub-culturing the test dilutions from the MIC experiment onto unseeded plates of Luria–Bertani agar. Subsequently, the plates underwent an additional incubation period of 18–24 h, and the MBC was defined as the highest dilution at which no individual bacterial colony was observed on the plates.

#### 4.3.4. Evaluation of Mycelial Growth

The antifungal effectiveness of the synthesized compounds was evaluated through the “mycelial growth test,” employing the “food poisoned” technique [48]. Standard potato dextrose agar (PDA) medium was employed for the experiment. Each test compound, dissolved in dimethyl sulfoxide to achieve a 1% (*w*/*v*) concentration, was dispensed in 0.1 mL (containing 1 mg of the compound) through a sterilized pipette into a sterile Petri plate. Subsequently, 20 mL of medium was added to the Petri plate, thoroughly mixed, and allowed to solidify. Inoculation took place at the center of each plate by placing a 5 mm mycelium block from each fungus. These mycelium blocks, extracted using a cork borer from the actively growing area of 5-day-old fungal cultures on potato dextrose agar, were inverted in the center of each Petri plate to maximize contact between the mycelium and the culture medium. The inoculated plates were incubated at 25 ± 2 °C. This experimental procedure was replicated three times, incorporating appropriate control plates (containing only potato dextrose agar without test compounds). After 5 days of incubation, the diameter of radial mycelial growth for each fungus was measured. The average of these three measurements was recorded as the radial mycelial growth diameter, expressed in millimeters (mm). The percentage inhibition of mycelial growth of the test fungus was calculated by the following equation:

I = (C − T)/C × 100

where “I” represents the percentage of inhibition, “C” stands for the diameter of the fungal colony in the control (DMSO), and “T” signifies the diameter of the fungal colony in the treatment.

### 4.4. Cytotoxic Activity Evaluation

The evaluation of the toxicity of derivatives of mannopyranoside was conducted using the brine shrimp lethality assay (BSLA) method, as outlined by reference [45]. The mannopyranoside derivatives being evaluated were dissolved in dimethyl sulfoxide (DMSO) and combined at four distinct concentrations: 20, 40, 80, and 160 µL. The intended result was achieved by adding 5 mL of sodium chloride solution to each vial. The vials designated A, B, C, and D contained items with concentrations of 4, 8, 16, and 32 µL, respectively. The experiment was conducted in triplicate for each dosage, wherein each vial was filled with a total of 10 brine shrimp nauplii. The mortality rate of nauplii was evaluated at each dosage level, and subsequently, the average percentage was calculated. No deaths were observed in the control group.

### 4.5. Structure Activity Relationship (SAR)

The study employed structure–activity relationship (SAR) analysis to make predictions regarding antibacterial activity, relying on the molecular structure of the pharmaceutical target. This technique is commonly utilized in the drug design process to aid in the discovery or development of new compounds with desirable properties. In this investigation, we conducted SAR studies, drawing inspiration from the membrane permeation hypothesis described by Hunt [49] and Kim [50].

### 4.6. Molecular Docking Studies

#### 4.6.1. Selection and Preparation of the Receptor

The molecules were specifically designed to target the H5N1 influenza virus, with the 6VMZ receptor chosen as the primary target. The 6VMZ receptor, in this context, represents the hemagglutinin of the H5N1 influenza virus. Within this receptor, the interacting ligand formed hydrogen bonds with ARG 26, ASP 302, GLU 469, and ALA 471, while hydrophobic interactions occurred with SER 297, ILE 301, and LYS 470. The receptor consists of three chains, each composed of 334 amino acids. Markedly, 88.8% of the receptor’s amino acids were found within the most favorable region, according to the Ramachandran plot (Figure S3). To prepare the receptor for computational analysis, we utilized Swiss PDB Viewer for energy minimization of the proteins. Subsequently, we assessed the receptors for missing residues using PyMol software (version 2.5) and ensured the correct ionization state of the amino acids within the receptor through the H++ server. Further analysis involved an evaluation of the surrounding residues of the cocrystallized ligands using Drug Discovery Studio. For the docking analysis and conversion of the PDB format into PDBQT format, we employed AutodockVina (version 1.2) and AutoDock Tools (ADT) (version 1.5.6) from the MGL software package (version 1.5.6), respectively.

#### 4.6.2. Ligand Preparation

The structural representations of the synthesized compounds were constructed using Avogadro software (version 1.2.0). Subsequently, the molecular geometry was optimized using the MMFF94 force field and the steepest descent method. Following optimization, three-dimensional coordinates were incorporated into the structures through the utilization of Open Babel software (version 2.4.1). To further refine the ligand’s conformation and energetics, a total energy minimization process was conducted employing Swiss PDB Viewer with the implementation of 20 steepest descent iterations. Subsequently, all polar hydrogens and Gasteiger charges were meticulously added to the molecules using AUTODOCK Vina (version 1.2). Finally, the molecular structures were saved in the PDBQT format for subsequent analyses.

#### 4.6.3. Molecular Docking Parameters of the Synthesized Compounds Interacting with 6VMZ

The dimensions of the grid box utilized for docking with the 6VMZ receptor in AutodockVina (version 1.2) were set as follows: −7.834 Å for the X-axis, −117,867 Å for the Y-axis, and −2.166 Å for the Z-axis. In total, approximately 25 conformational structures were generated as output for the ligand–protein docking experiments. Among these conformations, those exhibiting the most favorable binding energies were selected for further analysis. To gain insights into the docking results and investigate nonbonding interactions between the ligands and amino acid residues within the protein, Accelrys Discovery Studio (version 4.1) software was employed. This software facilitated the visualization and exploration of the docking outcomes [51].

### 4.7. Molecular Dynamic Simulation

Molecular dynamic simulations provided valuable insights into the atomic-level structural dynamics of the ligand molecules when interacting with the receptors. In this research, we employed the GROMACS 20.1 software package running on the LINUX UBUNTU platform to investigate the thermodynamic properties of the ligand–receptor complex. Prior to initiating the simulation study, several preparatory steps were undertaken. First, the ligand molecule was separated from the protein–ligand complex. Subsequently, GROMACS-compatible gro files were generated, incorporating the CHARMM 36 force field parameters. Next, TIP3P water molecules, solvent species, and sodium and chloride ions were introduced into the processed files. The processed files then underwent energy minimization utilizing the steepest descent algorithm over a 100 ns molecular dynamics (MD) simulation run. The energy minimization process was executed in two distinct phases. Initially, the number of particles, volume, and temperature (NVT) were held constant, followed by maintaining the number of particles, pressure, and temperature constant. Specifically, the simulation was conducted under a pressure of 1 ATM and at a temperature of 298 °K. The MD simulation trajectories were subsequently analyzed using the GROMACS 20.1 software. Key parameters, such as the root mean square deviation (RMSD) and root mean square fluctuation (RMSF) of the protein–ligand complexes, were computed using the gmx rms and gmxrmsf tools, respectively. Additionally, measurements of the solvent-accessible surface area (SASA) and the radius of gyration (Rg) were performed using the gmxsasa and gmx gyrate utilities, respectively. To visualize and present these trajectories graphically, Qtgrace software (https://sourceforge.net/projects/qtgrace/, accessed on 6 June 2023) was employed [52]. 

### 4.8. MM/PBSA Analysis

Molecular mechanics/Poisson–Boltzmann surface area (MM/PBSA) analysis determines the protein–ligand interaction energy after atomic level interaction. This comprehensive analysis encompassed the evaluation of various energy components, including van der Waal energy, electrostatic energy, polar solvation energy, solvent-accessible surface area (SASA) energy, binding energy, and the individual contributions of residues to the overall binding energies [53].

### 4.9. Frontier Molecular Orbital (FMO) Analysis

The electric and optical properties of the synthesized molecules were calculated using the FMO theory using basis sets B3LYP/6−31+G (d,p). The molecules exhibited zero charges and a single multiplicity. In FMO theory, the most crucial orbitals in a structure are the frontier molecular orbitals, comprising the highest occupied molecular orbital (HOMO) and the lowest unoccupied molecular orbital (LUMO). The HOMO, the outermost orbital occupied by electrons, acts as an electron donor, while the LUMO, the foremost vacant innermost orbital unoccupied by electrons, serves as an electron acceptor [54]. The electron-donating capability of a molecule is linked to its E_HOMO_, where a higher HOMO energy (smaller negative value) indicates an enhanced ability to donate electrons. Various parameters, including the energy gap (ΔE) between the HOMO and LUMO, softness, electronegativity, chemical hardness, and electrophilicity index values, were computed. The FMO analysis was conducted using GAMESS software (version 2023.R1), and data visualization was performed using WxMacMolPlt (version 0.9.58).

$$\mathrm{Chemical}\;\mathrm{hardness}\text{: }\eta =\frac{I-A}{2};\;\mathrm{softness}\;\zeta =\frac{1}{2\eta };\;\mathrm{electronegativity}\text{: }\mu =-\frac{I+A}{2};\;\mathrm{Electrophilicity}\;\mathrm{index}\text{: }\Psi =\frac{\mu 2}{2\eta },$$

where A and I are the electron affinity and ionization potential, respectively. A = −E_LUMO_ and I = −E_HOMO_.

### 4.10. Molecular Electrostatic Potential (MEP) Investigation

This investigation was used to identify the structural orientation and spatial arrangements of functional groups present in a chemical structure, and this contour map explained the importance of forces felt by a chemical structure due to its functional group and electron distribution. The MEP map provides information about the distribution of positive and negative charges in a molecule, which can be used to understand its chemical and physical properties [55]. For example, regions of high negative potential indicate areas of the molecule that are likely to attract positively charged species, while regions of high positive potential indicate areas of the molecule that are likely to attract negatively charged species. Overall, MEP map analysis is a powerful tool for understanding the electronic properties of molecules and predicting their behavior in chemical reactions and biological processes. In molecular electrostatic potential map, blue, green, yellow, orange, and red colors represent different potentials of nucleophilic and electrophilic attacks. It means potential of nucleophilic attacks decreases from blue region to red region. Also, the region for electrophilicity increases from green to red. The MEPs of the synthesized molecules were computed at the B3LYP functional and 6−31 + G(d,p) level of theory, and the data provide valuable evidence about the possible nucleophilic and electrophilic sites present in the structures [56].

### 4.11. OSIRIS Calculation

The OSIRIS software (version 2.9.1) was utilized for the in silico prediction and assessment of various molecular properties, including cLogP, solubility, molecular weight, topological polar surface area, drug-likeness, drug score, mutagenic risk, tumorigenic risk, irritant risk, and reproductive risk, for the synthesized molecules.

### 4.12. Pharmacokinetic Prediction

In the realm of drug development, the anticipation of the ADMET properties of potential compounds stands as a critical endeavor aimed at averting their clinical trial failures. To heighten the prospects of these compounds evolving into viable drug candidates, we subjected the most promising esters to comprehensive computational assessments leveraging tools such as pkCSM (pkCSM (uq.edu.au), accessed on 26 June 2023) [57]. These evaluations were chiefly centered on in silico pharmacokinetic parameters, encompassing an appraisal of the esters’ intestinal absorption capabilities in humans, their potential to traverse the blood–brain barrier and access the central nervous system, and a comprehensive comprehension of their metabolic alterations within the human body, total clearance rates, and toxicity profiles. Lipinski’s rule of five properties was obtained from the SwissADME server (www.swissadme.ch/index.php) (accessed on 23 June 2023).

### 4.13. Statistical Analysis

For every examined parameter, the experimental findings were depicted as the mean accompanied by the standard error, and these data were derived from three replicates. Statistical analysis was performed employing two-tailed Student’s *t* tests when applicable. Statistical significance was attributed solely to *p* values below 0.05.

## 5. Conclusions

In this study, a comprehensive analysis of mannopyranoside derivatives was conducted, encompassing both in vitro and in silico assessments of their antimicrobial, thermodynamic, molecular docking, molecular dynamics, and drug-like properties. The introduction of diverse aliphatic and aromatic groups into the mannopyranoside framework was found to exert a significant influence on their biological activity. Notably, octanoyl and benzenesulfonyl-substituted derivatives, specifically **4** and **6**, exhibited enhanced potential against bacterial organisms, accompanied by improved pharmacokinetic and biological profiles. These findings were substantiated by molecular docking studies, which unveiled promising antiviral activity of mannopyranoside derivatives against the influenza virus A (H5N1) receptor. Compounds **4** and **6** displayed robust binding interactions and favorable binding energies with the H5N1 receptor, indicative of their substantial in silico potential in combatting this virus. Furthermore, molecular dynamics simulations extending up to 100 ns corroborated the stability of the protein–ligand complex, affirming its resilience in biological systems. Additionally, these analogs underwent a thorough assessment of their pharmacokinetic properties, encompassing toxicity prediction, in silico OSIRIS prediction, and drug-likeness evaluations. Encouragingly, a majority of the designed molecules exhibited improved kinetic parameters while adhering to drug-likeness criteria. These results collectively suggest that these analogs hold promise as potential drug candidates. However, it is important to note that this study was conducted using synthetic, antimicrobial, and computational methods. Further validation through wet-lab experiments, both in vivo and in vitro, is imperative to ascertain the potential of these analogs as effective treatments against the H5N1 virus.

## Figures and Tables

**Table 1:** Structure of the synthesized mannopyranoside analogs **2**–**6**.

Entry	Chemical Structure
**2**	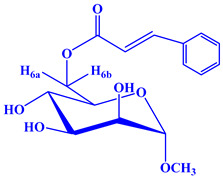
**3**	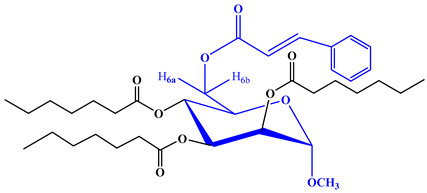
**4**	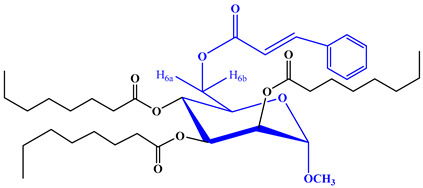
**5**	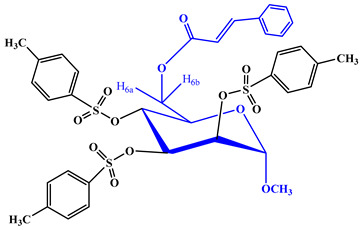
**6**	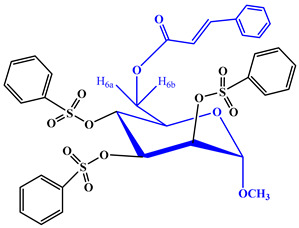

**Table 2:** Zone of inhibition observed against Gram-positive and Gram-negative bacteria by derivatives **1**–**6**.

Diameter of Inhibition Zone (mm)						
Entry	*B. subtilis* (+ve)	*S. aureus* (+ve)	*B. cereus* (+ve)	*E. coli* (−ve)	*S.typhi* (−ve)	*P. aeruginosa* (−ve)
**1**	NI	NI	NI	NI	NI	NI
**2**	NI	11.75 ± 0.3	07.11 ± 0.5	NI	12.41 ± 0.3	NI
**3**	NI	NI	NI	10.25 ± 0.2	NI	NI
**4**	10.51 ± 0.6	* 14.10 ± 0.7	* 15.17 ± 0.4	11.53 ± 0.2	10.23 ± 0.5	09.19 ± 0.6
**5**	12.15 ± 0.7	NI	08.10 ± 0.3	9.00 ± 0.2	NI	12.36 ± 0.2
**6**	10.13 ± 0.8	* 15.12 ± 0.1	* 15.15 ± 0.3	9.00 ± 0.3	9.17 ± 0.1	8.50 ± 0.2
Azithromycin	** 18.5 ± 0.3	** 17.75 ± 0.3	** 17.22 ± 0.3	** 17.25 ± 0.1	** 18.0 ± 0.2	** 18.5 ± 0.3

The data are presented as the mean ± SD, and the values are represented for triplicate experiments. Statistically significant inhibition (*p* < 0.05) is marked with an asterisk (*) for test compounds and a double asterisk (**) for the reference antibiotic azithromycin. NI = no inhibition.

**Table 3:** Antifungal activities of all synthesized derivatives in (%) of inhibition.

Entry	% Inhibition of Fungal Mycelial Growth in mm (20 μg/μL)	
	*Aspergillus niger*	*Aspergillus flavus*
**1**	NI	NI
**2**	NI	* 91.34 ± 1.0
**3**	NI	* 91.12 ± 1.0
**4**	* 96.22 ± 1.1	* 98.47 ± 1.0
**5**	57.62 ± 1.1	* 81.30 ± 1.0
**6**	NI	* 97.02 ± 1.1
Nystatin	** 65.4 ± 1.0	** 64.1 ± 1.0

The data are presented as the mean ± SD, and the values are represented for triplicate experiments. Statistically significant inhibition (*p* < 0.05) is marked with an asterisk (*) for test compounds and a double asterisk (**) for the reference antibiotic azithromycin. NI = no inhibition.

**Table 4:** Molecular docking studies data of the synthesized molecules against 6VMZ.

Entry	Receptors PDB id	Binding Energy (kcal/mol)	Interactive Residues with 4.0 Ǻ
**1.**	6VMZ	(−) 4.3	ARG 26, ASP 302, GLU 469, and ALA 471 by hydrogen bond interactions; SER 297, ILE 301, and LYS 470 by hydrophobic interactions.
**2.**		(−) 6.3	ARG 55 and GLN 311 by hydrogen bond interactions; ALA 284, SER 283, SER 287, TYR 289, LEU 306, ASN 309, and SER 310 by hydrophobic interactions.
**3.**		(−) 6.9	ARG 305 by hydrogen bond interaction; SER 287, GLY 288, TYR 289, GLU 294, LEU 306, GLN 308, ASN 309, and LEU 466 by hydrophobic interactions.
**4.**		(−) 7.2	SER 287 and ARG 305 by hydrogen bond interactions; ALA 284, GLY 288, TYR 289, ASP 290, ARG 293, GLU 294, LEU 306, GLN 308, ASN 309, LEU 466, and SER 467 by hydrophobic interactions.
**5.**		(−) 4.7	ARG 305 by hydrogen bond interaction; GLY 288, TYR 289, ASP 290, ARG 293, GLU 294, LEU 306, GLN 308, ASN 309, LEU 466, and SER 467 by hydrophobic interactions.
**6.**		(−) 7.0	ARG 305 by hydrogen bond interaction; ALA 284, SER 287, TYR 289, GLU 294, LEU 306, GLN 308, ASN 309, and LEU 466 by hydrophobic interactions.

**Table 5:** MM/PBSA analysis data.

Entry	Van der Waal Energy (kJ/mol)	Electrostaticenergy (kJ/mol)	Polar Solvationenergy (kJ/mol)	SASA Energy (kJ/mol)	Binding Energy (kJ/mol)
**1**	−3.980	−2.911	−16.839	−0.775	−24.496
**2**	−20.233	−8.457	14.906	−3.076	−16.892
**3**	−0.037	0.196	17.849	0.106	17.783
**4**	−0.185	−158.346	−39.939	0.033	−198.299
**5**	−0.151	−1.282	24.136	0.181	22.887
**6**	−104.331	−23.951	113.680	−13.269	−27.877

**Table 6:** FMO analysis data of compounds **1**–**6**.

Entry	E_HOMO_ (eV)	E_LUMO_ (eV)	ΔE Gap (eV)	I	A	η	ζ	μ	Ψ
**1**	−7.34	0.217	7.13	7.34	0.217	3.56	0.14	3.77	1.99
**2**	−6.61	−2.04	4.57	6.61	2.04	2.28	0.21	4.32	4.08
**3**	−6.77	−2.50	4.27	6.77	2.50	2.13	0.23	4.63	5.02
**4**	−6.83	−2.93	3.90	6.83	1.95	2.49	0.25	4.88	6.10
**5**	−6.61	−2.61	4.00	6.61	2.00	2.35	0.25	4.61	5.31
**6**	−6.12	−2.85	3.27	6.12	2.85	1.64	0.31	4.48	6.13

**Table 7:** Pharmacokinetics properties.

Entry	Water Solubility (log mol/L)	Caco-2 Permeability	Intestinal Absorption	Skin Permeability
**2**	−1.673	0.31	63.236	−3.437
**3**	−4.937	0.784	75.372	−2.729
**4**	−4.408	0.748	77.499	−2.733
**5**	−3.822	−0.216	85.269	−2.735
**6**	−3.781	−0.439	84.047	−2.735

**Table 8:** Prediction in silico of pharmacokinetics study.

Entry	Metabolism	Distribution			Execration
	CYP2C19 Inhibitior	Vdss (log L/kg)	BBB Permeability	CNS Permeability	Total Clearance
**2**	No	−0.668	−0.848	−3.643	0.473
**3**	No	−0.404	−1.858	−2.81	1.559
**4**	No	−0.522	−1.927	−2.694	1.609
**5**	No	−0.343	−2.814	−3.575	0.175
**6**	No	−0.446	−2.778	−3.726	0.384

**Table 9:** OSIRIS analytical data.

Entry	Toxicity Risk				Clog P	Solubility	M. Weight	TPSA	Drug Likeness	Drug Score
**1**	Mutagenic	Tumorigenic	Irritant	Reproductive effective	−2.19	0.12	194	99.38	−7.28	0.49
**2**	−ve	−ve	−ve	−ve	−0.15	−6.59	792	193.8	−8.47	0.09
**3**	−ve	−ve	+ve	−ve	0.06	−1.83	324	105.4	−5.32	0.28
**4**	−ve	−ve	+ve	−ve	9.7	−7.92	702	123.6	−30.7	0.04
**5**	−ve	−ve	+ve	−ve	10.66	−8.74	758	140.7	−25.1	0.04
**6**	−ve	−ve	+ve	−ve	−1.18	−5.56	724	193.8	−7.09	0.11

## Data Availability

Data are available in this article and the Supplementary Materials.

## Supplementary Materials

The following supporting information can be downloaded at https://www.mdpi.com/article/10.3390/molecules28248001/s1, Figure S1: FMO analysis data of compounds **1**, **2**, **3**, **4**, **5**, and **6**; Figure S2: MEP analysis data of compounds **1**, **2**, **3**, **4**, **5**, and **6**; Figure S3: Ramachandran plot of 6VMZ.

## Abbreviations

ADMET	Absorption, distribution, metabolism, excretion, and toxicity
DFT	Density functional theory
HOMO	Highest occupied molecular orbital
LUMO	Lowest unoccupied molecular orbital
MD	Molecular dynamics
MEP	Molecular electrostatic potential
PASS	Prediction of substance activity spectra
SAR	Structure–activity relationship
FTIR	Fourier-transform infrared
MRSA	Methicillin-resistant *Staphylococcus aureus*
RMSD	Root mean square deviation
RMSF	Root mean square fluctuation
SASA	Solvent-accessible surface area
MM/PBSA	Molecular mechanics/Poisson–Boltzmann surface area
FMO	Frontier molecular orbital
