# Supplementary material for: Exploring Cinnamoyl-Substituted Mannopyranosides: Synthesis, Evaluation of Antimicrobial Properties, and Molecular Docking Studies Targeting H5N1 Influenza A Virus
Source: Molecules. 2023 Dec 7;28(24):8001. doi: 10.3390/molecules28248001 (PMC10745968; doi:10.3390/molecules28248001)
Supplement: Supplementary file 1 [file molecules-28-08001-s001.zip › molecules-2703470-supplementary.pdf]

Supplementary Materials

Exploring Cinnamoyl-Substituted Mannopyranosides: Synthesis, Evaluation of Antimicrobial Properties, and Molecular Docking Studies Targeting H5N1 Influenza A Virus

Sabina Akter [1], Bader Y. Alhatlani [2,*], Emad M. Abdallah [3], Supriyo Saha [4], Jannatul Ferdous [1], Md Emdad Hossain [5], Ferdausi Ali [6] and Sarkar M. A. Kawsar [1,*]

[1] Laboratory of Carbohydrate and Nucleoside Chemistry (LCNC), Department of Chemistry, Faculty of
Science, University of Chittagong, Chittagong 4331, Bangladesh; sabinaprimu@gmail.com (S.A.);
jannat_080911@yahoo.com (J.F.)

[2] Unit of Scientific Research, Applied College, Qassim University, Buraydah 52571, Saudi Arabia

[3] Department of Science Laboratories, College of Science and Arts, Qassim University, ArRass 51921, Saudi Arabia; 140208@qu.edu.sa

[4] Uttaranchal Institute of Pharmaceutical Sciences, Uttaranchal University, Dehradun 248007, Uttarakhand, India; supriyo9@gmail.com

[5] Wazed Miah Science Research Centre, Jahangirnagar University, Savar, Dhaka 1342, Bangladesh; emdad121542@gmail.com

[6] Department of Microbiology, Faculty of Biological Science, University of Chittagong, Chittagong 4331, Bangladesh; seema@cu.ac.bd

* Correspondence: balhatlani@qu.edu.sa (B.Y.A.); akawsarabe@yahoo.com (S.M.A.K.)

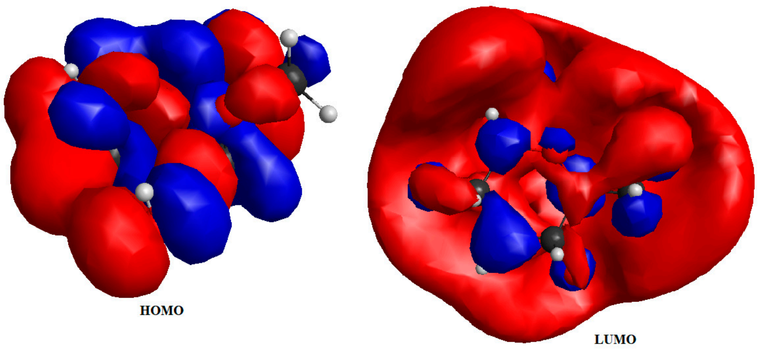

1

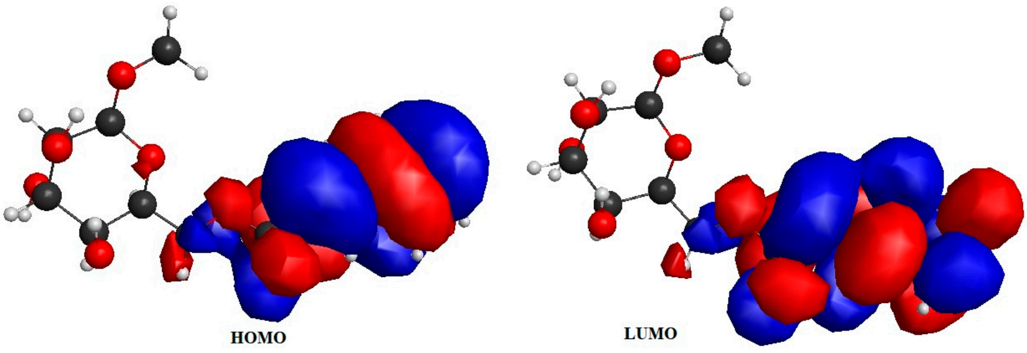

2

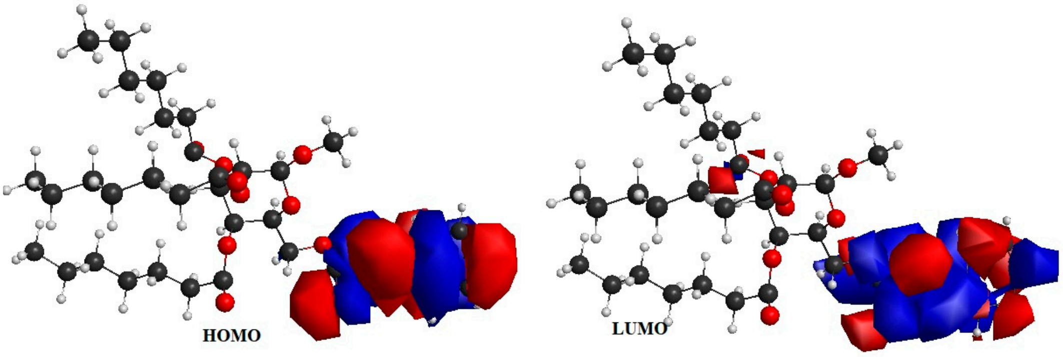

3

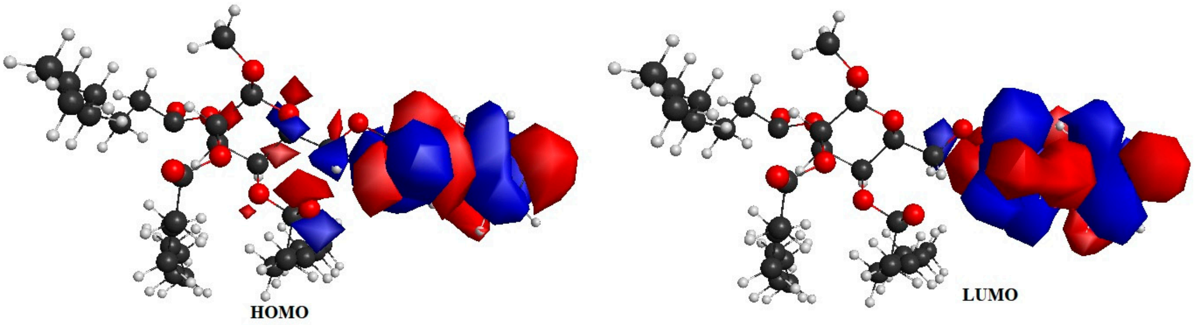

4

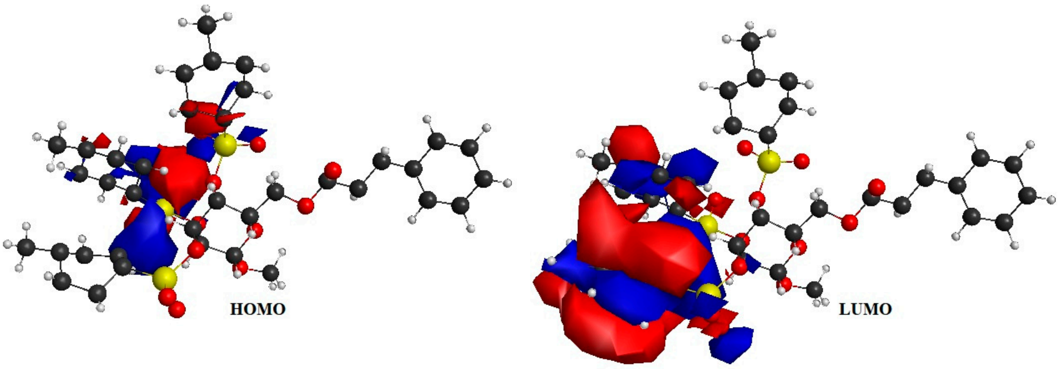

5

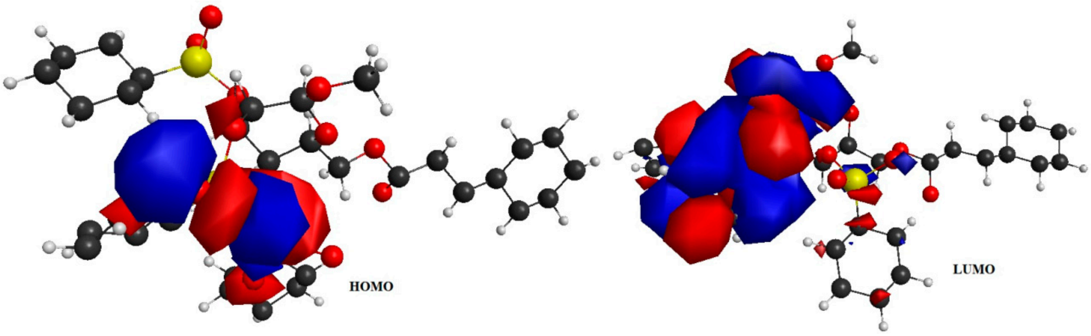

6

Figure S1. FMO analysis data of compounds **1, 2, 3, 4, 5,** and **6**.

Fixed Range -0.2 to 0.2
Click again for dynamic scale

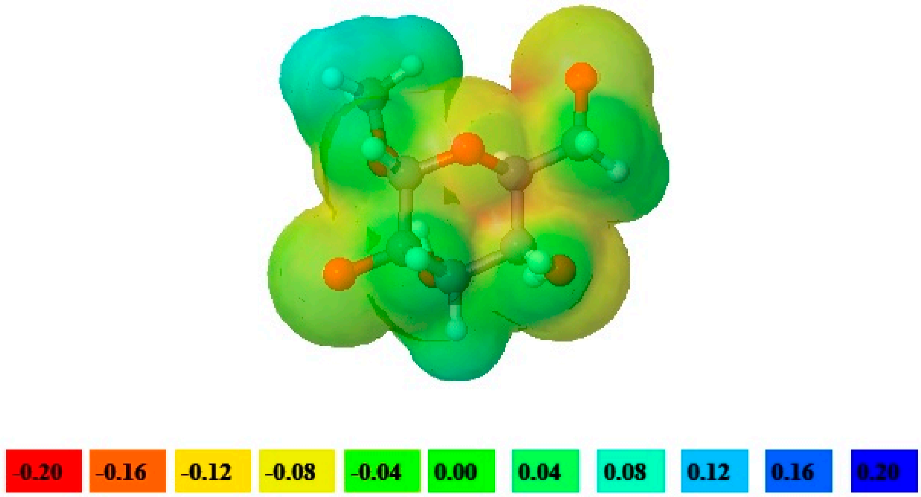

1

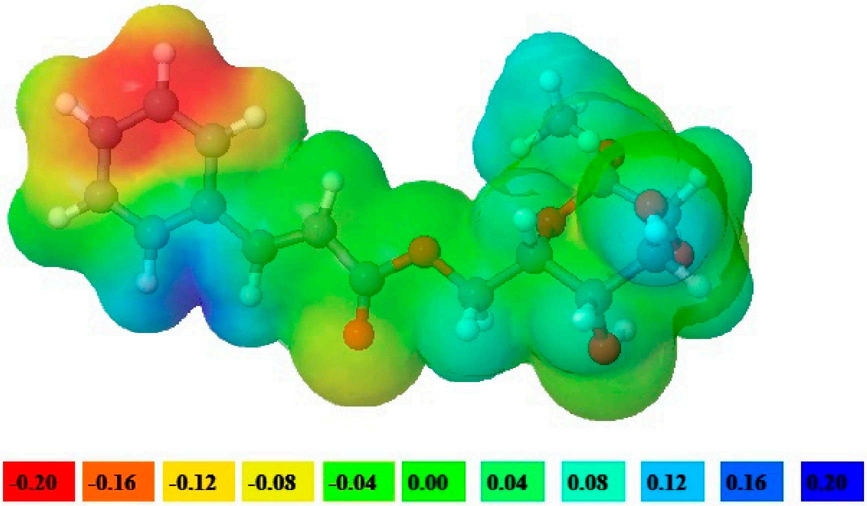

2

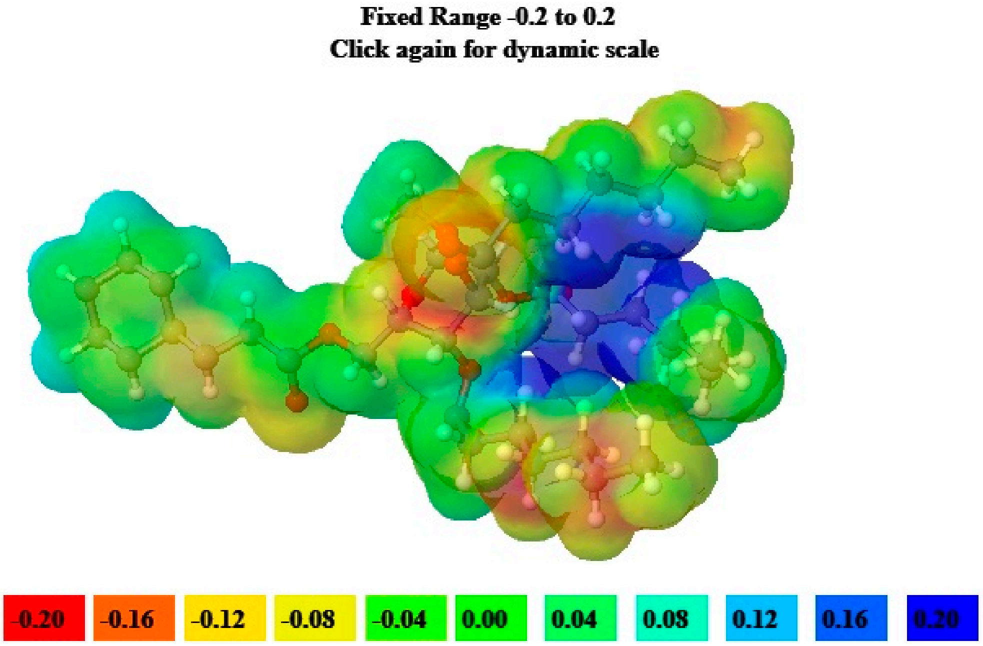
Fixed Range -0.2 to 0.2
Click again for dynamic scale
-0.20
-0.16
-0.12
-0.08
-0.04
0.00
0.04
0.08
0.12
0.16
0.20

3

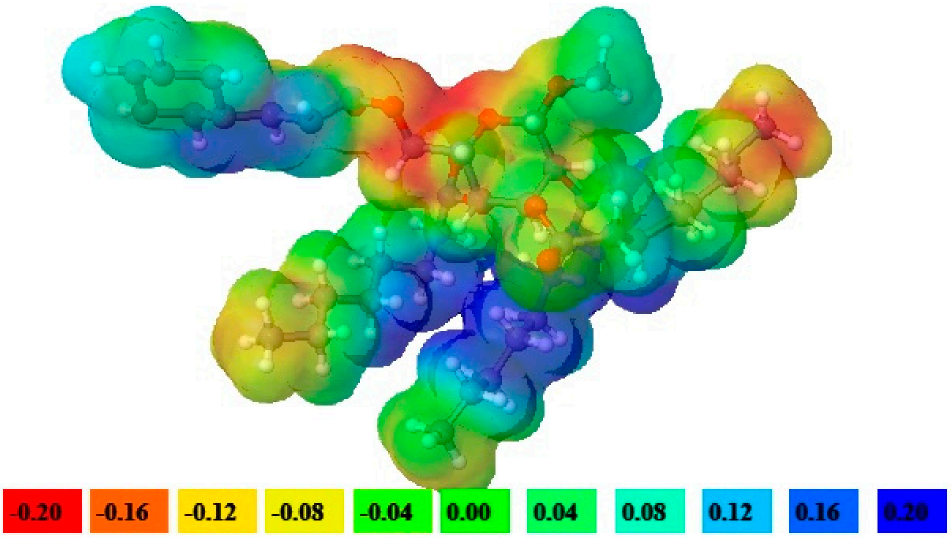
-0.20
-0.16
-0.12
-0.08
-0.04
0.00
0.04
0.08
0.12
0.16
0.20

4

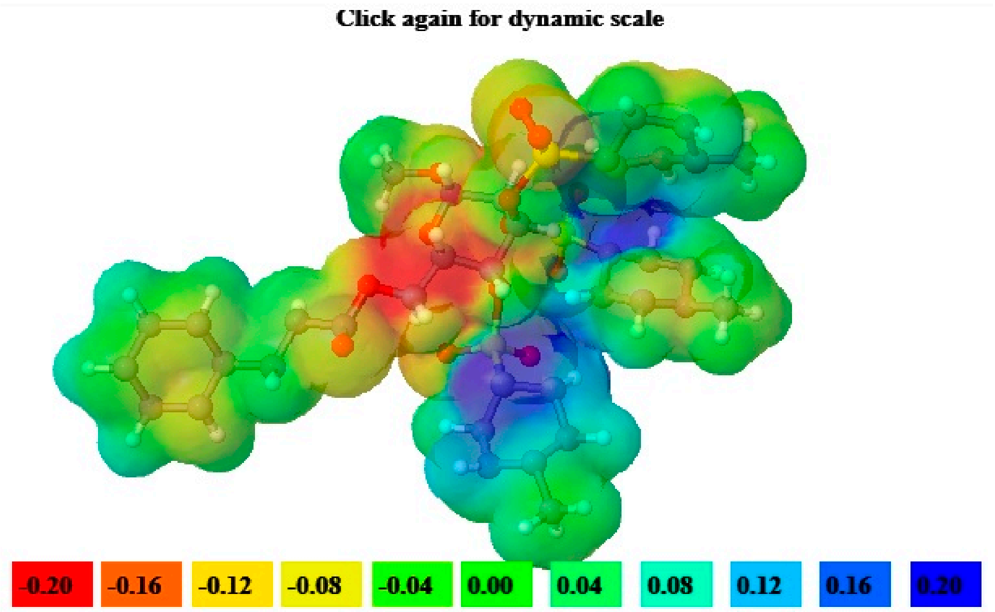

5

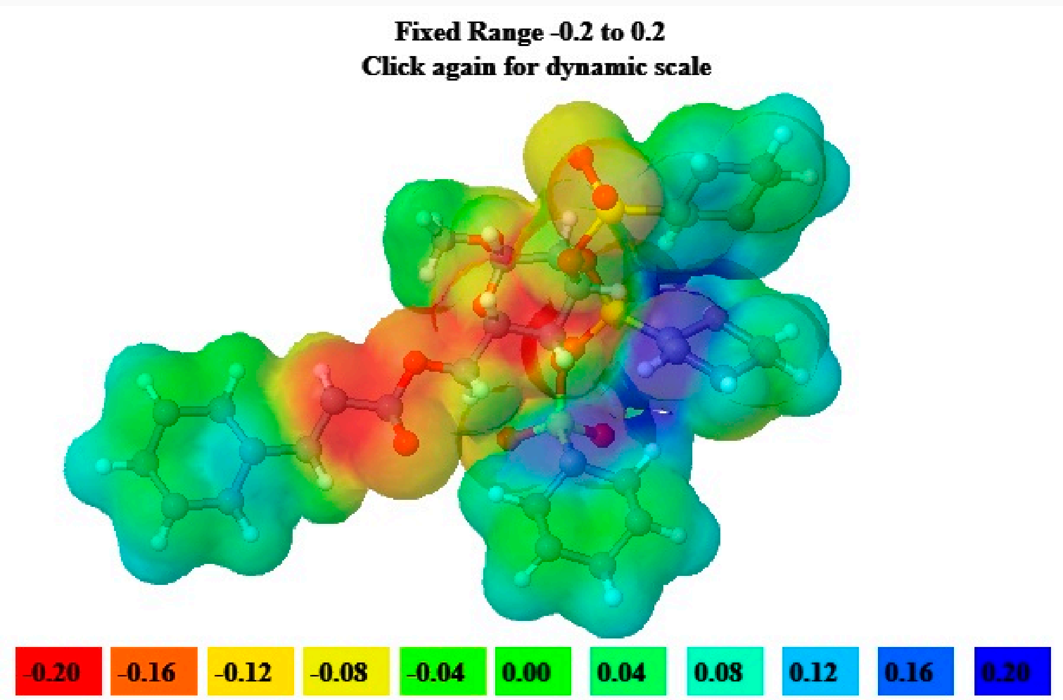

6

Figure S2. MEP analysis data of compounds **1, 2, 3, 4, 5,** and **6**.

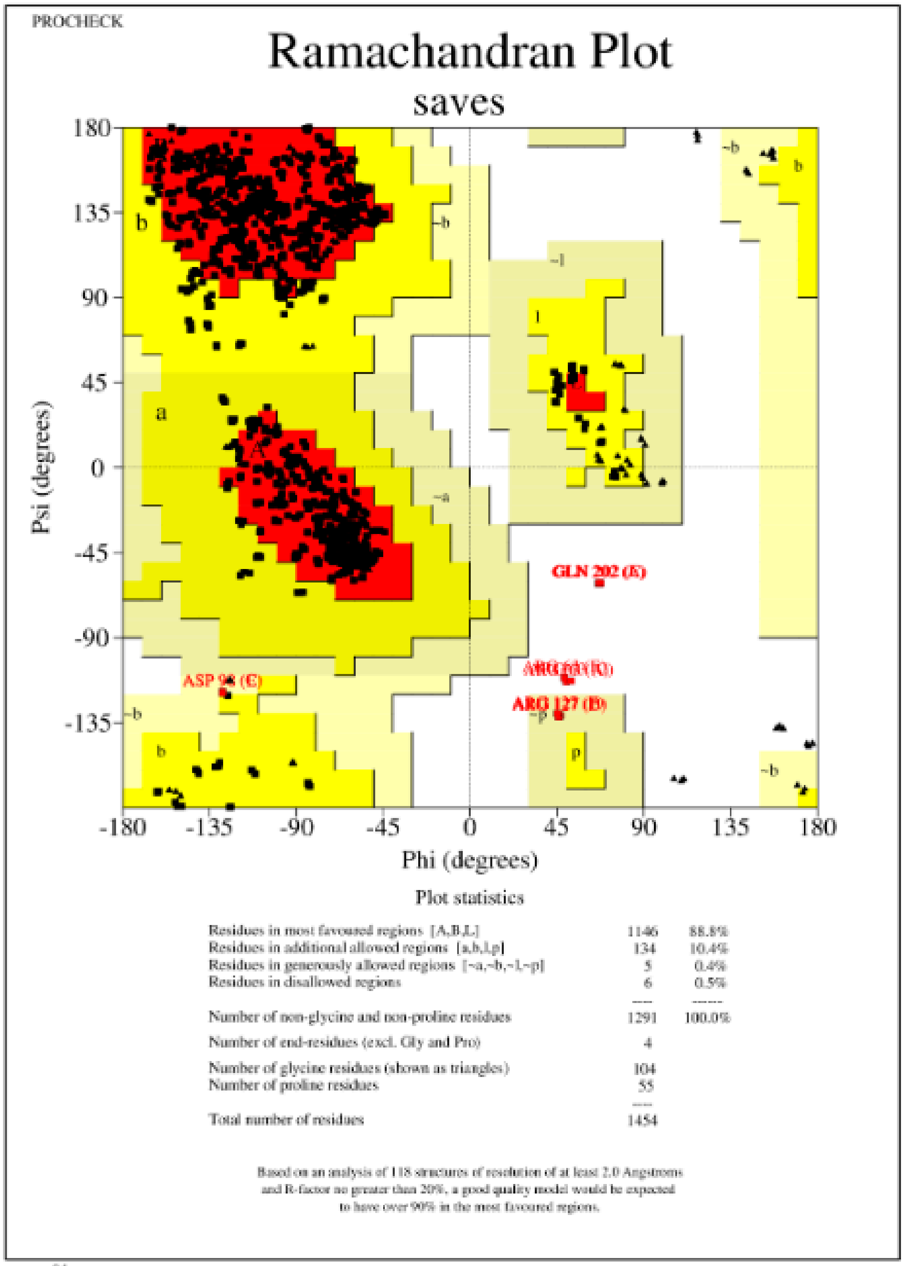

Figure S3. Ramachandran plot of 6VMZ.
